# Reliable Analysis of Single-Unit Recordings from the Human Brain under Noisy Conditions: Tracking Neurons over Hours

**DOI:** 10.1371/journal.pone.0166598

**Published:** 2016-12-08

**Authors:** Johannes Niediek, Jan Boström, Christian E. Elger, Florian Mormann

**Affiliations:** 1 Department of Epileptology, University of Bonn, Bonn, Germany; 2 Department of Neurosurgery, University of Bonn, Bonn, Germany; Consejo Superior de Investigaciones Cientificas, SPAIN

## Abstract

Recording extracellulary from neurons in the brains of animals in vivo is among the most established experimental techniques in neuroscience, and has recently become feasible in humans. Many interesting scientific questions can be addressed only when extracellular recordings last several hours, and when individual neurons are tracked throughout the entire recording. Such questions regard, for example, neuronal mechanisms of learning and memory consolidation, and the generation of epileptic seizures. Several difficulties have so far limited the use of extracellular multi-hour recordings in neuroscience: Datasets become huge, and data are necessarily noisy in clinical recording environments. No methods for spike sorting of such recordings have been available. Spike sorting refers to the process of identifying the contributions of several neurons to the signal recorded in one electrode. To overcome these difficulties, we developed *Combinato*: a complete data-analysis framework for spike sorting in noisy recordings lasting twelve hours or more. Our framework includes software for artifact rejection, automatic spike sorting, manual optimization, and efficient visualization of results. Our completely automatic framework excels at two tasks: It outperforms existing methods when tested on simulated and real data, and it enables researchers to analyze multi-hour recordings. We evaluated our methods on both short and multi-hour simulated datasets. To evaluate the performance of our methods in an actual neuroscientific experiment, we used data from from neurosurgical patients, recorded in order to identify visually responsive neurons in the medial temporal lobe. These neurons responded to the semantic content, rather than to visual features, of a given stimulus. To test our methods with multi-hour recordings, we made use of neurons in the human medial temporal lobe that respond selectively to the same stimulus in the evening and next morning.

## Introduction

Tracking single- and multi-unit activity over hours, possibly during sleep, allows to address important questions regarding neural mechanisms of learning and memory consolidation. For example, a popular theory posits that declarative memory consolidation during sleep depends on the re-activation of neuronal ensembles that were active during earlier behavior [1, 2].

Over the last years, it has become possible to record from hundreds of channels simultaneously [3–5], and modern recording systems allow to record continuously for hours and days, producing datasets that are typically hundreds of gigabytes in size.

Many spike sorting algorithms have been evaluated in the past [5–11]. The datasets used in these studies were usually simulations or recordings characterized by stationary (constant) noise levels, absence of non-neural artifacts, and short duration.

Even though spike sorting algorithms perform well on small datasets, tracking the activity of individual neurons over many hours has remained a major challenge: spike sorting algorithms have to be computationally efficient to deal with spike counts in the order of hundreds of thousands per channel, must account for both slow and sudden changes in spike waveform, and have to cope with periods of excessive signal contamination, as inevitable in multi-hour recordings in clinical settings. Furthermore, current spike sorting methods often require manual optimization, a time-consuming task in the case of multi-hour recordings.

Here, we present and evaluate *Combinato*: A software framework for unsupervised spike sorting of noisy long-term recordings. The core of our framework is a novel spike sorting algorithm based on block-wise iterative superparamagnetic clustering (SPC; [12]). This core algorithm is accompanied by methods for artifact rejection and tools for the visualization of results.

The importance of ground-truth data for the validation of spike sorting methods is becoming increasingly recognized [13]. We thus validated Combinato on a recently published dataset of simulated neural activity [14], showing that our method outperforms state-of-the-art spike sorting methods. This holds true even when the result of our *automated* spike sorting is compared to the published result of *manually optimized* spike sorting results on the same data [8]. When tested on simulated recordings lasting ten hours, our algorithms were capable of recovering on average 74.6% of the simulated neurons, despite drift and high noise levels in the simulations.

We also evaluated the performance of our method in a visual stimulus presentation experiment. The purpose of the experiment was to identify neurons that respond selectively and invariantly to visually presented stimuli [15]. Without any manual intervention, Combinato identified more neuronal responses than common spike sorting methods that require manual optimization.

Lastly, evaluation on eight whole-night recordings from the temporal lobes of epilepsy patients showed that our method tracks visually selective single- and multi-units over more than 12 hours.

An implementation of *Combinato* in Python is publicly available along with installation instructions and a user tutorial, available on GitHub (https://github.com/jniediek/combinato). This implementation is licensed under the MIT License.

## Design and Implementation

**Fig 1:**
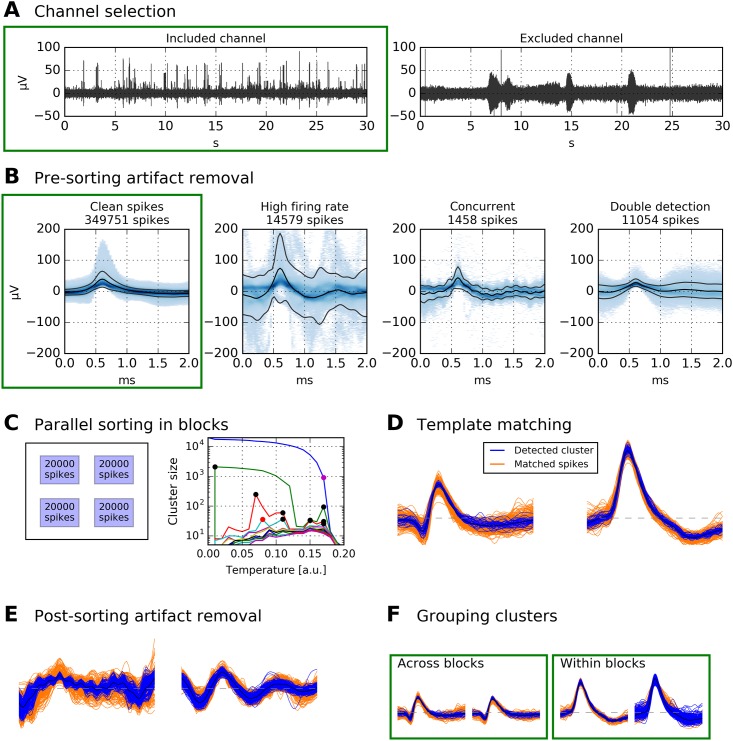
Schematic of data processing. Data displayed in green boxes are passed on to the subsequent stage. **A** Channels with no unit activity (i.e., broken or empty channels) are discarded. Displayed are bandpass-filtered recordings of two channels in the human MTL (passband 300 Hz to 1000 Hz). **B** After spike extraction, pre-sorting artifact rejection is performed. Displayed are density plots of all spikes extracted from a 12-hour recording (same channel as left panel in A). Artifact rejection removes ≈ 27 000 spikes, and ≈ 350 000 remain. **C** Clean spikes from B are split into blocks of 20 000. In this example, the 350 000 spikes are split into 18 blocks. All blocks are spike-sorted in parallel. For each block, clusters from several “temperatures” are selected. Displayed is an example for one block. Black dots in the temperature plot correspond to clusters that were selected, red dots to clusters that were not selected because their spikes had already been selected at lower temperatures, and the purple dot marks the highest temperature used. Large clusters are again subjected to iterative spike sorting. **D** Template matching is used to assign the remaining spikes to clusters. **E** Artifact clusters are removed. **F** Physiological clusters are grouped, both within each block and across blocks.

Before describing our spike sorting framework in detail, we will provide a brief outline of its structure; see Fig 1 for an illustration of individual steps. The first two steps are channel selection (Fig 1A) and spike extraction. These steps are conceptually independent from any specific spike sorting algorithm. The next step is pre-sorting artifact removal (Fig 1B), after which clean spikes are passed to block-wise iterative sorting (Fig 1C). Remaining spikes are then assigned by template matching (Fig 1D), and non-neural clusters are detected and removed (Fig 1E). The last step is to re-combine all clusters from the different blocks (Fig 1F).

### Channel selection and spike extraction

In a typical multi-channel setup, not all electrodes record meaningful signals. Reasons for corrupted signals include: bad/broken wiring in the electrodes, inadequate electrode impedance, excessive pick-up of 50/60 Hz power line noise and its higher harmonics. Furthermore, in recordings from the human brain, a micro-electrode bundle sometimes has its tip in white matter or cerebrospinal fluid, where recording of action potentials is impossible.

**Fig 2:**
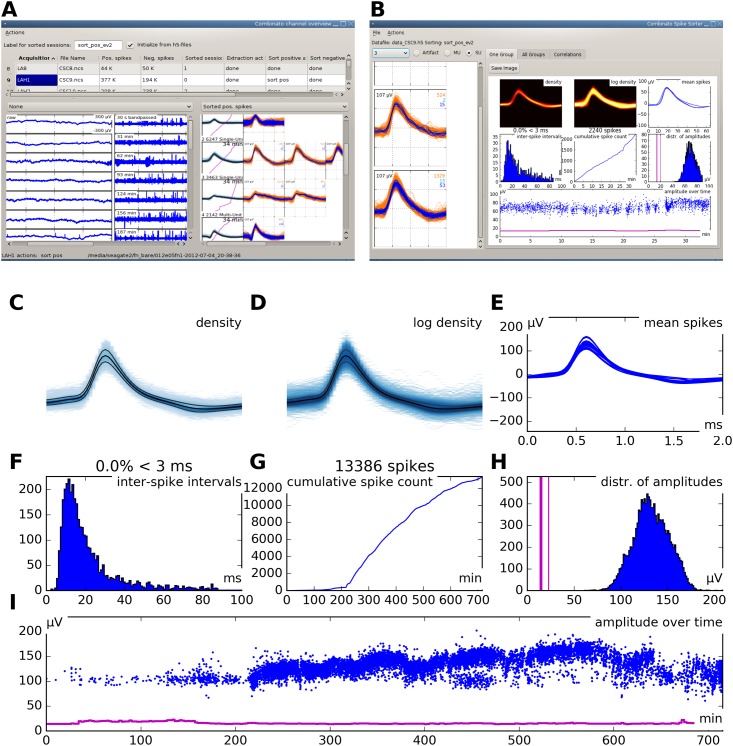
Graphical user interface. **A** Screenshot of the graphical user interface (GUI) used for channel selection. Raw and filtered data traces of all channels in a recording session are displayed along with spike sorting results from every channel for which sorting has already been performed. **B** Screenshot of the GUI used for visualization and manual optimization of spike sorting results. The interface shows several informative statistics for one unit. The individual elements are explained in panels C through I. **C** Density plot of all spike waveforms within a cluster group. **D** Same as C, but using a logarithmic scale. **E** Overlay plot of all mean spike waveforms in a group of merged clusters. **F** Histogram of inter-spike intervals. **G** Cumulative spike counts over time (700 minutes in this example). Note that the unit in this example appears to become more active after the first 200 minutes of recording. Detailed inspection of the other cluster groups is necessary to decide whether this is really the case or merely an effect of over-clustering and false re-grouping. **H** Distribution of spike maxima. The three vertical pink lines indicate the minimum, median, and maximum of the detection thresholds over time. Note that in this example, spike maxima are clearly separated from the detection threshold. **I** Spike amplitude maxima over time. The pink line is the extraction threshold. Note that the extraction threshold is relatively stable, while the maxima show considerable drift.

To exclude unsuitable channels from subsequent analysis, our analysis framework contains a viewer program. The program displays segments of each channel’s signal at different temporal resolutions (for a screenshot, see Fig 2A).

Viewing the data at different time scales allows to assess presence of action potentials, contamination by electrical noise, and contamination by low-frequency artifacts. Examples of one selected and one discarded channel are shown in Fig 1A.

Spikes are detected and extracted from all selected channels by a standard method similar to WaveClus [6], for details see Section A in S1 Text.

### Pre-sorting artifact rejection

Long-term recordings, especially from human subjects in a clinical setting, inevitably contain periods of excessive noise, e.g. due to subject movement or electrically interfering medical equipment. During such periods, recordings are typically contaminated by events of non-neural origin. We detect such artifacts both before and after spike sorting. Before spike sorting, we use the following procedure, the parameters of which can be modified according to specific demands:

*(1) Removal of time periods exceeding reasonable neuronal firing rates.* For each recording channel, event counts are calculated in time-bins of 500 ms, with an overlap of 250 ms. Time-bins containing more than 100 events (corresponding to a firing rate of 200 Hz) are excluded from further processing.

*(2) Events exceeding a certain amplitude.* All events exceeding a threshold of 1 mV are excluded.

*(3) Removal of overlapping detections.* Because our spike detection algorithm does not impose any artificial refractory period, two extracted spikes can overlap. This happens when the interval between two subsequent threshold crossings is shorter than the extraction window (typically around 2 ms); for example, when there is sinusoidal electrical noise in the range of 2 kHz or when two different neurons fire action potentials with a very short lag. Depending on the respective scientific question, keeping both waveforms might be desirable (e.g. when analyzing synaptic coupling). When two detections occur within 1.5 ms, our default is to keep the waveform with the larger maximum, and discard the other one.

*(4) Events occurring concurrently on many channels.* Movement artifacts and periods of excessive electrical noise typically occur simultaneously on many channels, whereas an action potential is typically recorded on one channel only. We thus partition the spike times extracted from all channels into time-bins of 3 ms, with an overlap of 1.5 ms, and count, for each bin, the recording channels with at least one event. A time-bin is excluded if it contains an event on 50% or more of the channels.

Note that criteria *(1)* to *(3)* are applied independently on individual channels, whereas *(4)* takes into account all recording channels at the same time.

### Segmentation and spike sorting in blocks

After the exclusion of artifact events, the remaining spikes are segmented into independent blocks, such that each block consists of *N*_block_ consecutive spikes (by default, *N*_block_ = 20 000; see Table 1 for complete parameter values). These blocks are then spike-sorted independently using parallel processing to make efficient use of modern multi-core computers. The algorithm that processes each block is based on superparamagnetic clustering (SPC; [12]) of wavelet coefficients, as introduced to spike sorting in WaveClus [6]. However, our procedure differs from WaveClus in the following ways: clusters at several different “temperatures” of the SPC algorithm are selected automatically; clusters are re-clustered in an iterative procedure; similar clusters are merged automatically; template matching is performed at two different stages; unassigned spikes are iteratively re-clustered. What follows is a detailed description of our per-block algorithm.

**Table 1:** Relevant parameters for the proposed spike sorting framework along with their default values.

Name	Description	Default value
*N*_block_	Number of spikes per sorting block	20 000
*C*_max_	Maximum number of clusters at one temperature	5
*S*_min_	Minimum number of spikes in a cluster	15
*R*_min_	Minimum cluster size for iterative clustering	2000
*N*_rep_	Number of clustering iterations	1
*f*_1_	Radius for within-block template matching	0.75
*f*_2_	Radius for across-blocks template matching	3
*C*_stop_	Threshold at which merging of clusters stops	1.8

*Feature selection.* Similar to WaveClus, a four-level wavelet decomposition is computed for each spike using Haar wavelets. This yields an (*n* × *k*)-array of wavelet coefficients, where *n* is the number of spikes and *k* the number of sampling points. To reduce feature dimensionality, we select, out of these *k* dimensions, the 10 dimensions in which the distribution of wavelet coefficients differs most from normality, as quantified by the Kolmogorov–Smirnov test statistic [6].

*Clustering.* The 10 selected wavelet coefficients are passed on to superparamagnetic clustering (SPC). SPC depends on a parameter *T* (called “temperature” due to its motivation from statistical physics). For each value of *T*, SPC partitions the input data in a particular way. Our default is to use *n*_*T*_ = 21 different values for *T*, equally spaced in [0, 0.2]. We use these independent *n*_*T*_ data partionings in a combined way to select data clusters.

The idea is to iterate through all temperatures from low to high: at each temperature *T*_*j*_ the clusters present at *T*_*j*_ are sorted by size (i.e., number of spikes). Then, the *i*-th largest cluster at *T*_*j*_ is selected for later processing if it is larger than the *i*-th largest cluster at surrounding temperatures *T*_*j*−1_ and *T*_*j*+1_. During this iteration over the temperatures, a unique cluster identification number is assigned to each selected cluster, and all spikes belonging to a selected cluster are marked as its members. Importantly, all spikes already marked at lower temperatures *T* < *T*_*j*_ are not reassigned, but remain members of the clusters selected earlier.

In other words, local maxima of “cluster size” as a function of “temperature” are selected for later processing, and spikes are irreversibly assigned to clusters by moving from low to high temperatures.

The following constraints apply: *(1)* A maximum number of *C*_max_ clusters are selected at any given temperature. *(2)* A cluster needs to contain at least *S*_min_ spikes in order to be selected, where *S*_min_ is defined either as an absolute number or as a fraction of the total number of spikes. *(3)* The cluster assignment procedure begins at the second temperature only, where local maxima are defined. Typical values for *S*_min_ and *C*_max_ are discussed in the Results section.

In this way, at each temperature up to *C*_max_ clusters are read out from SPC, such that each cluster contains at least *S*_min_ spikes. The cluster selection at different temperatures is illustrated by Fig 1C (right panel). Note that typically a large fraction of spikes is not assigned to any cluster at all, which makes a subsequent template matching step necessary.

*Splitting of large clusters.* Making use of all available temperatures considerably reduces the chances that a generated cluster contains spikes of two or more neurons (so-called *under-clustering*). To further reduce the risk of under-clustering by splitting clusters into sub-clusters, SPC clustering is run once again on every cluster that contains at least *R*_min_ spikes (by default, *R*_min_ = 2000).

*First template matching.* After clusters have been generated as outlined above, template matching is used to assign the yet unassigned spikes to existing clusters. For each cluster, its mean spike waveform is calculated, along with a measure of its total variance, $s:=\sqrt{\sum_{i=1}^{N}\text{var}\left(x_{i}\right)}$, where *var*(*x*_*i*_) denotes the variance of the cluster at the *i*-th sampling point. Then, the Euclidean distance between each spike and every cluster is calculated. Each spike is assigned to the cluster closest to it, provided the distance is smaller than *f*_1_ ⋅ *s*. Here *f*_1_ is a factor that controls the radius around a mean waveform where template matching is possible, in units of the variability of that cluster. By default, we use the conservative value *f*_1_ = 0.75 for this step because another template matching step is applied later, when pooling clusters from all blocks.

*Re-iteration.* After this first template matching step, the clustering procedure can be re-iterated on all spikes that are still not assigned to any cluster. This iteration of clustering and template-matching can be repeated *N*_rep_ times, but on our data, such iterations were unnecessary (hence by default *N*_rep_ = 1, see Results).

### Template matching across blocks

After all blocks of spikes from one channel have been independently spike-sorted, template matching is applied across blocks to assign the remaining unclustered spikes, using the same algorithm as for the within-block template matching. Here, we use *f*_2_ = 3. Spikes that are still not assigned to any cluster remain in a special “residual” cluster.

### Post-sorting artifact rejection

Although our pre-sorting artifact detection algorithm removes large fractions of non-neural events before spike sorting, it is still desirable to decide for each cluster whether it corresponds to neuronal activity or to residual noise in the recording. Often, artifact events appear in a stereotypical manner, e.g. in the case of sinusoidal electrical noise (see right panel of Fig 1E for an example of such an artifact cluster).

Our algorithm designates a cluster as non-neural if it meets any of the three following criteria based on its mean waveform. *(1)* The mean waveform has more than 5 local maxima. *(2)* The ratio of the largest local maximum to the second largest local maximum is less than 2, where only maxima separated by at least 0.3 ms are considered. *(3)* The amplitude range covered in the second half of the mean waveform is greater than the global maximum.

Furthermore, the standard error of the mean at each sampling point is calculated across all spikes in the cluster. A cluster is designated as an artifact if the mean of these standard errors across all sampling points is greater than 2 μV.

### Merging of clusters from all blocks

At this stage of processing, each block of spikes has been spike-sorted independently, remaining spikes have been assigned by template matching, and artifact clusters have been identified. The next step is to create groups of clusters belonging to the same unit. The input to this merging procedure is the pool of all non-artifact clusters from all blocks. The merging procedure has two aims: First, after spike sorting, two or more clusters in one block often correspond to the same unit (resulting from so-called *over-clustering*). Merging highly similar clusters within one block reduces the amount of over-clustering. Second, the same unit typically appears across many blocks (in sufficiently stable recordings, the same units should appear in *all* blocks). Merging highly similar clusters across blocks ensures that units can be tracked over the entire duration of the recording. Guided by empirical testing, we decided to perform both merging procedures—within and across blocks—in parallel. We use a simple hierarchical clustering method for cluster merging, based on the Euclidean distance between mean spike waveforms of the cluster groups. After merging the two clusters whose distance is minimal, the mean waveform is updated, and distances are re-calculated. Merging stops once the minimal distance is greater than a predefined threshold *C*_stop_ (by default, *C*_stop_ = 1.8). Importantly, our algorithm saves the original cluster identity of each spike, so that cluster grouping can later be undone if desired. It is also possible to use this cluster identity as a feature for subsequent analyses. Examples of cluster merging both within and across blocks are shown in Fig 1F.

### Optional manual verification

Our spike sorting framework comes with a graphical user interface (GUI), which is used to visualize sorting quality, to modify the grouping of clusters, and to mark additional artifact clusters, if necessary. Fig 2B shows a screenshot of the GUI. Different cluster visualization features are explained in Fig 2C through 2I. Some of these features are inspired by a recent publication [16]. The sparsely firing unit used as an example in Fig 2C through 2I was tracked over the time course of 700 minutes. The total number of spikes in this recording was approximately 330000. More detailed instructions on how to use the GUI are contained in S3 Text.

## Results

Our method proved useful both for spike sorting of short recordings (up to one hour), and for long-term tracking of unit activity over many hours. To demonstrate the broad applicability of our framework, we evaluated it using four different datasets: *(1)* simulated model data (simulated recording duration 10 minutes), *(2)* simulated model data with drift (simulated recording duration 10 hours), *(3)* short recordings from a visual stimulus presentation experiment, *(4)* whole-night recordings from epilepsy patients.

### Validation on simulated model data

It is becoming increasingly recognized that in order to estimate the reliability of spike sorting methods, using data with ground-truth is necessary [13]. To evaluate our method in a setting where ground truth is available, we used a recent dataset of simulated neural activity. Details regarding this dataset have been published [8] and the data are available online (http://bioweb.me/CPGJNM2012-dataset) [14]. The dataset consists of 95 simulations, each one representing 10 minutes of continuous recording, sampled at 24 kHz. Each simulation contains the activity of 2 to 20 neurons, superimposed on background noise and multi-unit activity. There are 5 simulations for each number of neurons, resulting in a total of 95 simulations. We chose this dataset because the performance of expert operators of WaveClus on it has been evaluated [8]. Using this dataset, we analyzed our algorithm’s reliability by comparing its performance to ground truth, as well as to expert operators’ results.

In a first step, spike detection was performed on the simulated dataset. Averaged over the 95 simulations, only 79.2% (SD 8.3%) of all spikes were found, which is indicative of the noise in the simulations (same numbers with WaveClus).

We then used our spike sorting method to spike-sort the extracted spikes. No post-sorting artifact detection was performed because the simulations did not contain any artifacts. We spike-sorted the first of the 95 files several times with various settings to empirically determine suitable parameters based on visual inspection of the spike sorting results, and then used these parameters to evaluate our method on the remaining 94 simulations. We used only one simulated channel for parameter optimization in order to avoid overfitting of parameters. For a more complete evaluation of the various parameters, see the following subsection.

**Fig 3:**
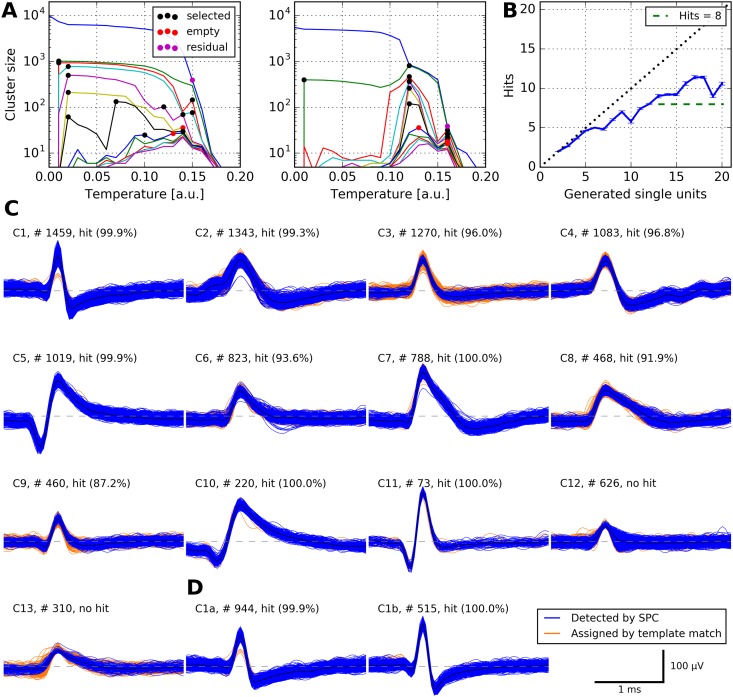
Performance of our algorithm on simulated data. **A** Cluster sizes at different temperatures for one of 95 simulations (simulation_5). Each marked location corresponds to an automatically selected cluster; up to *C*_max_ = 7 clusters are selected at each temperature. Left panel, input to this clustering step were all spikes in one simulated channel. Right panel, input to this clustering step were all spikes not assigned to any cluster during the first clustering step. **B** Performance of our algorithm on all simulated datasets. Each simulated dataset contained action potentials from 2 to 20 neurons. For each simulation, we calculated the number of *hits*: a unit *U* generated by our spike sorting method was considered a hit if at least 50% of the spikes in *U* belonged to one neuron and at least 50% of the spikes of that neuron were in *U*. Displayed is the number of hits as a function of the number of neurons in the simulations (error bars denote s.e.m.). Note that our algorithm is capable of detecting more than eight neurons, a typical maximum for manual operation of WaveClus [8]. **C** All automatically generated clusters from simulation_5. Shown are spike counts and the percentage of spikes in the detected unit that actually belonged to the corresponding neuron in the simulation. Eleven clusters were hits, two clusters were no hits. Note that cluster C11 was perfectly detected despite its low firing rate of 0.12 Hz. **D** Undoing an automatic merge in cluster C1 with our graphical interface generated another hit.

We used the following parameter values: *C*_max_ = 7; *C*_stop_ = 1.6; *N*_rep_ = 2; *R*_min_ = 1000; all other parameters were kept at their default values (see Table 1 for description of parameters). The deviations from default in *C*_max_, *C*_stop_, and *N*_rep_ reflect the relatively large number of true clusters in the simulated data, and the change in *R*_min_ accounts for the short overall duration of 10 minutes. We used our algorithm in its completely automatic mode without any manual interaction to strictly avoid a bias of any sort. Fig 3A shows samples of temperature plots with selected clusters marked.

To quantify the success of spike sorting, we used the same score as in [8]: A given unit *U* is considered a *hit* if it fulfills the following two criteria: *(1)* at least 50% of the spikes in *U* belong to one neuron; *(2)* at least 50% of the spikes of that neuron are in *U*. See Fig 3C for an example where our algorithm automatically generated 13 clusters, 11 which of were hits.

We calculated the number of hits for each of the 95 simulations in the test set. We then grouped the datasets by the number of neurons present, and calculated the fraction of hits for each group (cf. Table 1 in [8]).

Our algorithm significantly outperformed manual expert operators. In its completely automatic mode, it generated 71.5% (SD 13.8%) hits (percentage of simulated neurons), while the authors of [8] achieve 66.7% (SD 18.1%) on the same data with experts manually operating WaveClus (*T* = 31.5, *P* = 0.033, Wilcoxon signed-rank test).

Restricting analysis to the more difficult group of simulations with at least 8 neurons, our algorithm generated 64.5% (SD 8.2%) hits, while [8] achieved 58.9% (SD 14.1%) hits (*T* = 12.0, *P* = 0.034, Wilcoxon signed-rank test).

We visualized the proportion of hits for each number of neurons present in the simulations in Fig 3B (cf. Fig 4 in [8]).

We also verified that the number of hits can be further increased by manually optimizing sorting using our graphical interface. Fig 3D shows an example where manually undoing an automatic merge of two clusters produced an additional hit.

#### Evaluation at different parameter settings

**Fig 4:**
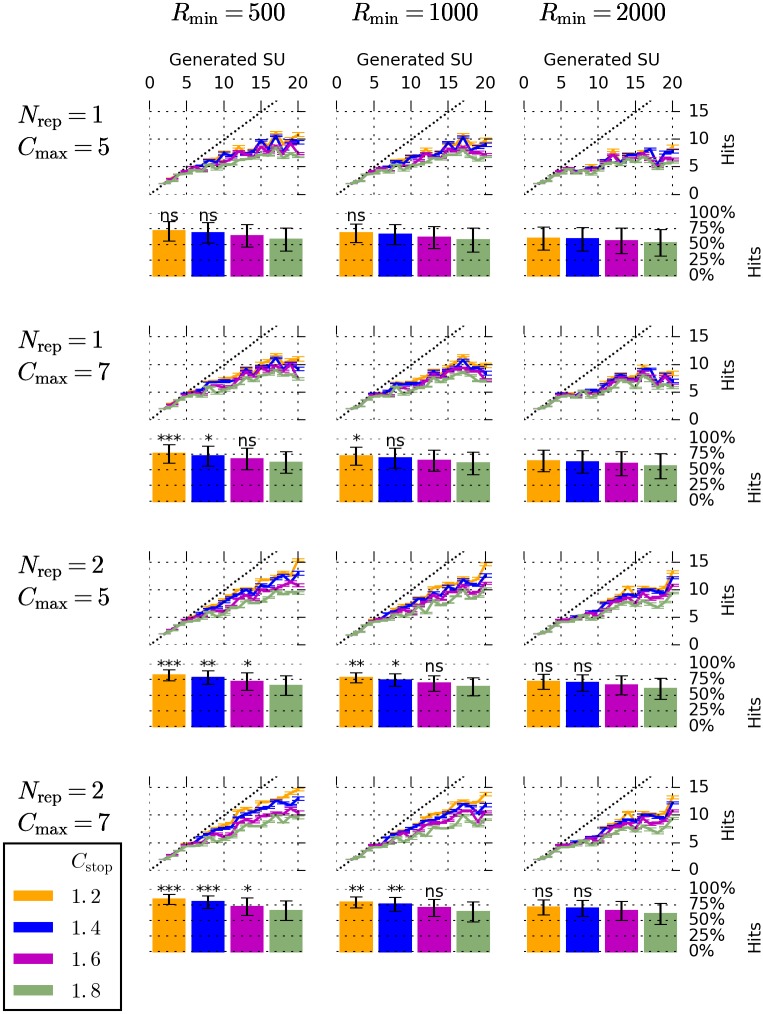
Performance of our algorithm on simulated data at different parameter settings. Results for a total of 48 different parameter settings are displayed. Each column of panels corresponds to one value of *R*_min_ (indicated above each column), and each row of panels corresponds to one pair of values for *N*_rep_ and *C*_max_ (indicated left of each row). Colors correspond to four different values of *C*_stop_, as indicated by the legend in the lower left. Each line plot shows the number of hits as a function of the number of neurons in the simulation (error bars denote s.e.m.), compare Fig 3B. Each bar plot represents the number of hits as a fraction of the number of neurons in the simulation (error bars denote standard deviation). The presence of asterisks or ‘ns’ above each bar indicate that the fraction of hits obtained at this particular choice of parameters is higher than the one obtained by manual expert operators in [8] (‘ns’ if *P* ≥ .05; * if *P* < .05; ** if *P* < .01; *** if *P* < .001). A Wilcoxon signed-rank test was used for all comparisons. Bars without ‘ns’ or asterisks indicate parameters at which the fraction of hits was lower than the one obtained by manual expert operators.

Having evaluated the performance of our algorithm with one parameter setting, we systematically investigated the influence of the parameters on the sorting result. We analyzed the same simulated dataset using a total of 48 different parameter settings. Specifically, we tested all combinations of the following: *C*_max_ ∈ {5, 7}, *R*_min_ ∈ {500, 1000, 2000}, *N*_rep_ ∈ {1, 2}, *C*_stop_ ∈ {1.2, 1.4, 1.6, 1.8}. For each of these settings, we quantified the success of spike sorting by counting the number of hits in each simulation. The results are displayed in Fig 4. We used a Wilcoxon signed-rank test in order to compare the number of hits at each parameter setting to the number of hits obtained by manual expert operators in [8]. Our method outperformed manual operators for 24 of the 48 different settings used, and in 13 settings, this difference was statistically significant.

At the ranges tested here, *R*_min_, *C*_stop_, and *N*_rep_ monotonically influenced the number of hits generated: Increasing *C*_stop_ above 1.2 always led to a decrease in the number of hits with all other parameters fixed (12 cases). The same was true when we increased *R*_min_ above 500 (16 cases), and when we changed *N*_rep_ from 2 to 1 (24 cases).

The highest fraction of hits was obtained for the setting *C*_max_ = 7, *R*_min_ = 500, *N*_rep_ = 2, and *C*_stop_ = 1.2. At this setting, 83.6% (SD 8.0%) hits (percentage of simulated units) were generated by our methods (manual operators in [8]: 66.7%, *T* = 3.0, *P* = 0.0003, Wilcoxon signed-rank test). The percentage of hits at the best of the 48 parameter settings tested was higher than at the parameter setting we had found by manually optimizing one simulated channel only (83.6% versus 71.5%). At the best of the 48 parameter settings, when including only the more difficult group with at least 8 neurons, our algorithm generated 80.0% (SD 5.8%) hits (manual operators in [8]: 58.9%, *T* = 0, *P* = 0.001, Wilcoxon signed-rank test).

#### Evaluation on multi-hour simulations

To test our algorithm’s capabilities at tracking neurons over many hours, we concatenated the simulated data from [8] after extracting spikes. For each number of simulated neurons, ranging from 2 to 20, we chose the first simulation containing this specific number of neurons and concatenated it 60 times, resulting in a total of 19 simulations of 10 hours duration each. In order to make the spike sorting task more difficult, we applied two modifications to each concatenated dataset: *(1)* We simulated electrode drift by multiplying the extracted spikes with a factor that linearly increased from 1 to 1.5 over the course of the 10 hours. *(2)* After this scaling, we added Gaussian noise to each datapoint of the extracted spikes. The noise had a mean of zero and a standard deviation of 20% of the maximum value attained in each simulation.

**Fig 5:**
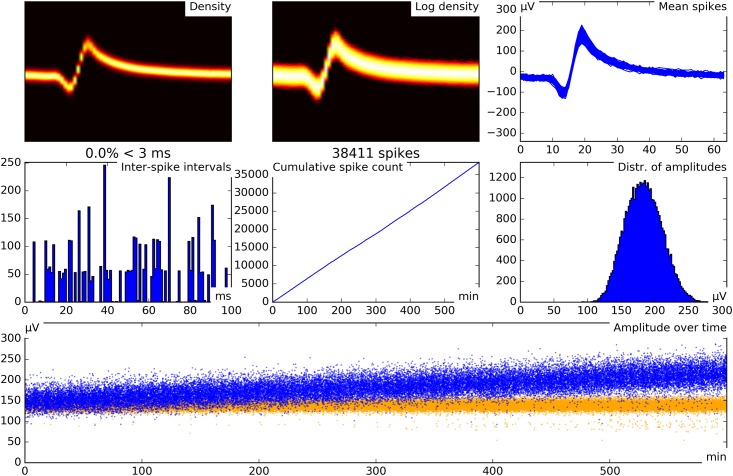
Example of a simulated neuron successfully tracked over 10 hours. This specific simulation was created by concatenating simulation_10 60 times, resulting in a total of 820800 spikes. Drift was simulated by multiplying the extracted spikes by a linearly increasing factor. Gaussian noise was added to the extracted spikes before sorting. The tracked unit has 38 411 spikes. Of these, 38 012 spikes belong to one unit in the simulation (which consists of 40 200 spikes), and 399 spikes belong to different units. The unit was successfully tracked despite the drift. Each subpanel is labelled according to its content. In the panel *Amplitude over time*, the tracked unit is displayed in blue, and the same unit without drift and without added noise is displayed in orange for comparison. Drift and added noise are clearly visible in the tracked unit.

We then used our algorithm on the concatenated, modified simulations. We tested four different parameter settings: Informed by the systematic parameter evaluation discussed above, we used *C*_max_ = 7, *R*_min_ = 500, and *N*_rep_ = 2. The parameter *C*_stop_ was set to 1.2, 1.4, 1.6, and 1.8, respectively. An example of a simulated unit that was successfully tracked over 10 hours is shown in Fig 5.

**Fig 6:**
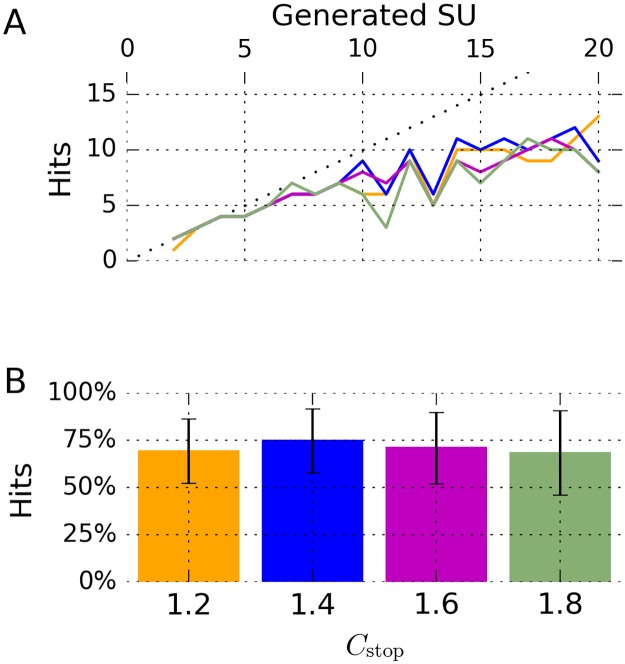
Performance of our algorithm on multi-hour simulated datasets. **A** Results for a total of 4 different parameter settings are displayed. The number of hits is shown as a function of the number of neurons in the simulations. See **B** for color legend. **B** Each bar represents the mean number of hits as a fraction of the number of neurons present in the simulation. Error bars denote standard deviation. The colors of the bars correspond to the lines in **A**.

Fig 6 shows the number of hits obtained at each setting and number of neurons. The highest number of hits, obtained at *C*_stop_ = 1.4, was 74.6% (SD 17.1%).

### Validation on a picture presentation experiment

After validating our method’s performance on simulated data, we then turned to data recorded from human subjects during a cognitive paradigm. Six epilepsy patients were implanted bilaterally with micro-electrodes in the medial temporal lobes (MTLs), for details see Section B in S1 Text. We used a picture presentation paradigm to screen for neurons in the MTL that responded selectively and possibly invariantly to a small number of visual stimuli. In these “screening sessions”, the six patients were presented with 130 to 150 pictures (mean 138.3) of well-known persons, landscapes, animals, and other objects. Pictures were presented in a pseudorandomized order on a laptop screen. Each picture was presented six times, for a duration of one second. Details of this paradigm have been described previously [15, 17, 18].

We used our viewer program (see Fig 2A) to exclude recording channels that clearly carried no unit activity. Starting from an initial total number of 536 channels, this left us with 409 recording channels (range 36 to 87 per session, median 70) from the amygdala, hippocampus, entorhinal cortex, and parahippocampal cortex.

**Fig 7:**
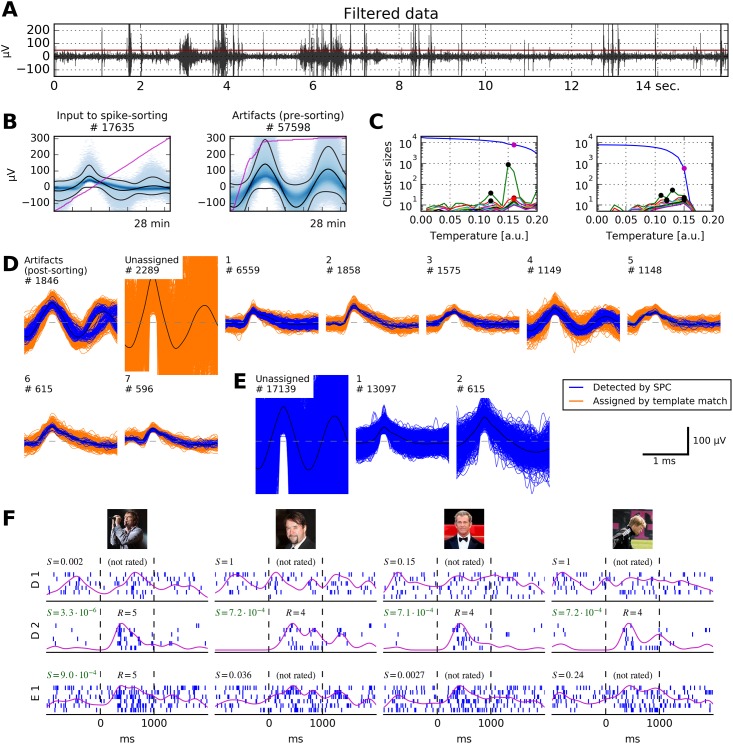
Our algorithm applied to recordings from the human medial temporal lobe (MTL). **A** 15 seconds of bandpass filtered data (passband 300 Hz to 1000 Hz) from a micro-electrode in the right anterior hippocampus. Extraction threshold is marked in red. Several artifact events are clearly visible. **B** This recording channel is extremely noisy: Our pre-sorting artifact detection removed ≈ 77% of all spikes from the recorded data. The pink lines depict the cumulative count of events over the course of the recording (28 minutes). **C** Cluster sizes at different temperatures. Left panel, input to the first clustering step were all non-artifact spikes. Right panel, input to the second clustering step were residual spikes not assigned to any cluster in the first clustering step. Color code of marked dots as in Fig 3. **D** Output of our sorting algorithm. Post-sorting artifact detection correctly identified several artifact clusters, but missed one (number 4). Six non-artifact clusters remain. **E** Result that expert operators generated manually with WaveClus. Two clusters were identified, ≈ 17000 spikes were left unassigned. **F** Results of the picture presentation experiment. Displayed are raster plots corresponding to Clusters 1 and 2 from D, and to Cluster 1 from E. Responses to four different pictures are shown. It is clearly visible that Cluster D 2 responds sharply to pictures of four male celebrities, while the responses of Cluster E 1 to the second, third and fourth picture are barely recognizable. No other cluster from D responded to any stimulus. S, score of the response; R, rating given to the response by human raters (see main text for details). Stimulus pictures displayed here have been replaced by similar pictures for legal and privacy reasons. Copyright notes: **F**.1 Photographer: Armin Kübelbeck, CC BY-SA 3.0, Wikimedia Commons (https://commons.wikimedia.org/wiki/File:Campino_02.jpg) **F**.2 “Mel Gibson at the Cannes film festival” by Georges Biard is licensed under CC BY-SA 3.0, Wikimedia Commons (https://commons.wikimedia.org/wiki/File:Mel_Gibson_2011_cropped.jpg) **F**.3 cropped from “German actor Jan Josef Liefers at the Cinema for Peace gala” by Thore Siebrands, licensed under CC BY 3.0, ipernity (http://www.ipernity.com/doc/siebbi/10852332) **F**.4 cropped from “Letztes Training von Olli Kahn beim FC Bayern München” by Dirk Vorderstraße, licensed under CC BY 2.0, Flickr (https://www.flickr.com/photos/dirkvorderstrasse/10560731386).

We used our software in its completely automatic mode to extract spikes, remove artifact spikes, sort spikes, and mark artifact clusters. The pre-sorting artifact rejection removed different fractions of spikes on different channels: 6.3% of all spikes were removed, but on some channels, up to 76.6% of the spikes were removed. See Table 2 for details and Fig 7 for an example of a channel on which clean clusters and clear neuronal responses were detected only after our pre-sorting artifact algorithm removed large amounts of noise. Fig 7D also shows examples of a correct post-sorting artifact rejection, as well as an artifact cluster that the post-sorting artifact criterion missed (Cluster 4 in Fig 7D). Table 2 and Fig 7 also give an impression of both the amount and characteristics of noise in the data used here.

**Table 2:** Effect of pre-sorting artifact removal.

Artifact type	% chan. affected	% spikes removed		
		mean over all chan.	max. over affected chan.	of all spikes
Firing rate	6.4	13.1	67.2	2.3
Amplitude	30.1	0.5	3.7	0.1
Double detection	96.3	8.7	48.6	3.7
Concurrent	64.3	3.5	41.2	0.3
Any of the above	96.6	12.0	76.6	6.3

These values show the variability of artifacts across channels. For example, only 6.4% of all channels were affected by the artifact criterion related to high firing rates, but on these affected channels, an average of 13.1% and a maximum of 67.2% of the spikes were removed. See main text for a detailed description of different types of artifacts.

**Fig 8:**
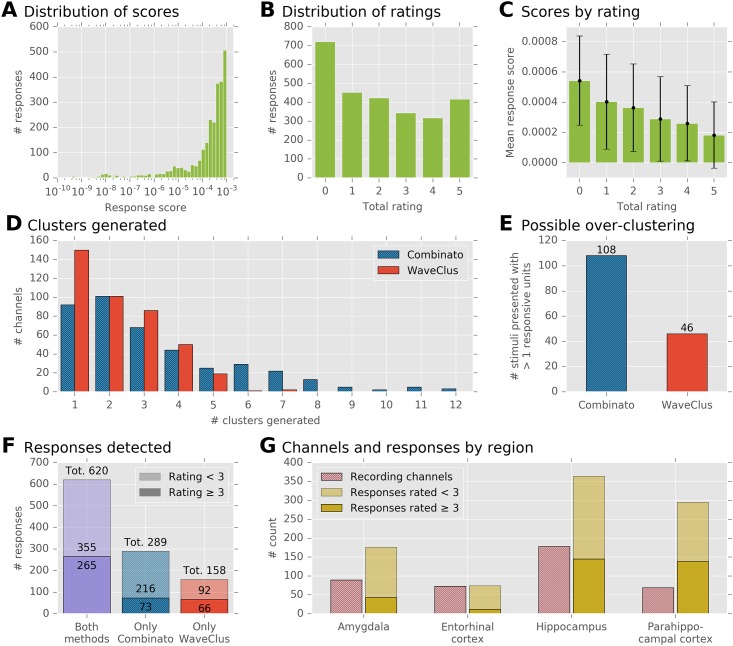
Evaluation of our algorithm on a visual stimulus presentation experiment. **A** Distribution of response scores. Some scores are extremely small, but the majority of scores lies in the interval [10^−4^, 10^−3^] (1939 out of 2672; 72.6%) **B** Each response’s rating is defined as the sum of the binary votes of five human raters. Of all 2672 ratings, 1596 (59.7%) were < 3, and 1076 (40.3%) were ≥ 3. **C** Mean score and standard deviation of responses at each rating. The relationship between mean scores and ratings is strictly monotonic, but the variance of scores at each rating is large. **D** Histogram of the numbers of clusters that were generated, on the same recordings, by Combinato and WaveClus. On average, Combinato generated more clusters. **E** Analysis of possible over-clustering. Displayed is the number of stimuli for which a response was detected in more than one cluster of the same recording channel. **F** Total numbers of detected responses. The numbers were corrected for possible over-clustering: only one response was counted per stimulus and channel, even if the response was detected in multiple clusters. Of all responses, 620 were detected both by Combinato and WaveClus. An additional 289 responses were detected only by Combinato, and further 158 responses only by WaveClus. The opaque parts of the bars correspond to responses that were rated 3 or better by expert raters. **G** Distribution of recording channels and responses across regions. Opaque parts of the bars as in F.

To test the performance of our automated algorithm against an established standard, we again chose WaveClus. We asked trained operators to analyze the same dataset: operators sorted the data with WaveClus and optimized sorting results manually using its graphical interface. These operators were uninformed about the analyses described here. Manual operation of WaveClus typically resulted in fewer units per channel than application of our automated method, see Fig 8D. We controlled for this difference in further evaluations (see below, and Discussion section).

We tested to which extent the single- and multi-unit responses found by manual spike sorting with WaveClus could be recovered by our automated method.

In the following, we use the term *WaveClus sorting* to refer to the clusters generated by manual operation of WaveClus, and *Combinato sorting* to refer to the clusters generated by our automated method.

To identify response-eliciting pictures and the corresponding single- or multi-units, we used a simple *response score* algorithm [17–19]. Briefly, action potentials from all six repetitions of a picture presentation (6 × 1000 ms) were binned into 19 overlapping time-bins with a duration of 100 ms each and an overlap of 50 ms. The Mann–Whitney U test was applied to each time-bin separately against a baseline distribution consisting of all 500 ms intervals preceding all picture onsets. These 19 p-values were subjected to the Benjamini–Hochberg procedure [20], yielding one single score. The combination of a neuronal cluster and a stimulus picture was then called a *response* if the p-value obtained by this procedure was below 0.001, and if action potentials were fired during at least four presentations of the picture.

We calculated response scores for all stimuli and all clusters from both sortings. For every channel, we included its unsorted multi-unit activity as an additional cluster. The response score algorithm yielded a total of 2672 numerically identified responses. Fig 8A shows the distribution of all response scores.

Our aim was to compare how WaveClus and Combinato perform in finding responsive neurons. Therefore, we could have compared how many numerically identified responses both methods yielded (WaveClus, 1274; Combinato, 1398). However, this comparison neglects two important aspect stemming from potential over-clustering: First, if Combinato tended to over-cluster the data, would a simple response count not be inflated due to responses from single neurons that would appear in multiple clusters because of over-clustering? Second, would a higher number of clusters not increase the number of detected responses simply because of false-positive detections? In light of these potential pitfalls, we used the following approach to ensure a fair comparison between WaveClus and Combinato.

We asked five expert human raters to rate each of the 2672 identified responses. For each response generated by the algorithm, raters were presented with a raster plot of the neural activity during the six picture presentations, showing spikes from one second prior to picture onset to one second after picture offset. Each rater was asked to assign a value of either one or zero to each raster plot, indicating whether or not it contained the typical pattern of a neuronal response. The total rating of the response was then defined as the sum of all raters’ scores, resulting in a number between zero and five. The raters were informed about the purpose of the procedure, but uninformed about the stimulus picture and clustering method that had generated each raster plot. As a measure of inter-rater agreement, we calculated Cohen’s *κ* for each of the ten pairs of raters [21]. The median *κ* was 0.466 (range 0.258 to 0.525).

Fig 8B shows the distribution of ratings. The rating procedure confirmed the high false-positive rate of the numeric response score: of all 2672 numerically detected responses, 1596 (59.7%) received a rating of less than 3, while 1076 (40.3%) were rated 3 or better. As expected, the mean numeric response score at a specific rating was a strictly decreasing function of the rating, see Fig 8C. Spearman’s rank correlation coefficient between the ranks and the scores was *ρ* = −0.399 (*P* = 10^−102^), and Kendall’s rank correlation coefficient was *τ*_*b*_ = −0.291 (*P* = 10^−112^).

We then used the ratings to control for possible over-clustering in the comparison of WaveClus and Combinato. First, if more than one cluster on a given channel responded to the same stimulus, we kept only the response that had received the highest rating, and dropped all others. This applied to 108 stimulus–channel combinations in the Combinato sorting and 46 stimulus–channel combinations in the WaveClus sorting, see Fig 8E. After removing all but one response for all of these stimulus–channel combinations, 778 responses remained in the WaveClus sorting, and 909 responses remained in the Combinato sorting. By applying this control, we ensured that no stimulus–channel combination could contribute more than once to these counts, which effectively ruled out response count inflations due to over-clustering. Fig 8F depicts the distribution of these responses: 620 responses were detected in both the Combinato and the WaveClus sorting, an additional 289 responses in the Combinato sorting only, and an additional 158 responses in the WaveClus sorting only.

Second, to ensure that the higher response count in the Combinato sorting was not just due to false-positive detections, we restricted our analyses to responses rated 3 or better. Here, 265 responses were detected in both sortings, 73 in the Combinato sorting only, and 66 in the WaveClus sorting only.

Responses were detected in all regions we recorded from. Fig 8G shows the regional distribution of responses as detected in the Combinato sorting.

A number of responses was detected by only one of the methods: 32% of the responses detected by Combinato were not detected by WaveClus, and 20% of the responses detected by WaveClus were not detected by Combinato. When including only responses rated 3 or better, 22% of the responses detected by Combinato were not detected by WaveClus, and 20% of the responses detected by WaveClus were not detected by Combinato.

**Fig 9:**
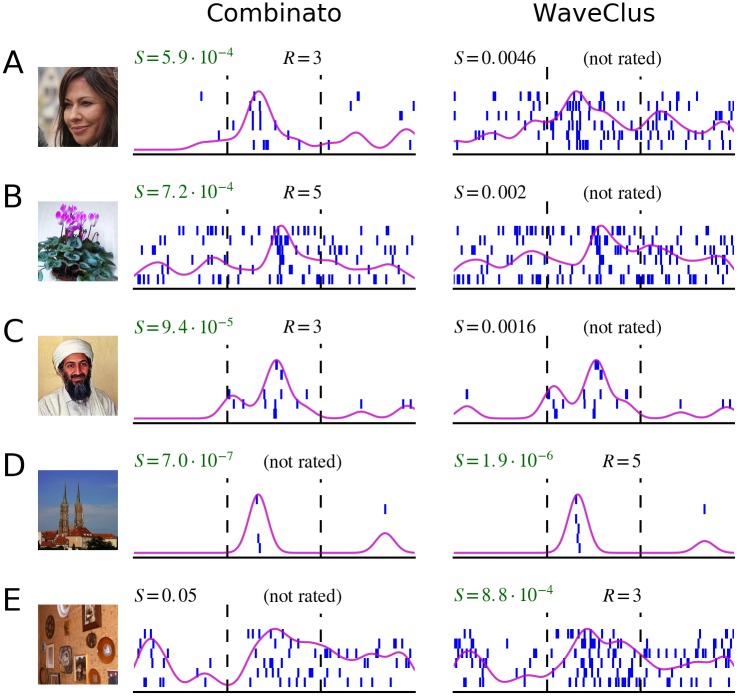
Responses detected by only one spike sorting algorithm. Displayed are five different visual stimuli, and corresponding neuronal responses. Each row (**A**–**E**) shows the visual stimulus presented and two raster plots. The raster plots on the left correspond to a unit in the Combinato sorting, and the raster plots on the right correspond to a unit in the WaveClus sorting, on the same channel. Differences in spike sorting become apparent. **A** Combinato generated a sparse unit that enabled detection of the neuronal response. The unit generated by manual operators of WaveClus was not detected as a response. **B**, **C** Tiny differences in the units’ composition led to a large difference in the numeric response score. **D** The unit generated by Combinato violates the requirement that one spike has to be fired during at least four picture presentations. **E** Differences in unit composition led to a large difference in the numeric response score. S, numeric score of the response; R, rating given to the response by human raters. Stimulus pictures displayed here have been replaced by similar pictures for legal and privacy reasons. Copyright notes: **A** Superbass, CC BY-SA 3.0, Wikimedia Commons (https://commons.wikimedia.org/wiki/File:Tatort_Keppler_Saalfeld.jpg)**B** “Violet” by J. Niediek is licensed under CC BY 4.0 **C** cropped from “Pakistani journalist Hamid Mir interviewing Osama bin Laden” by Hamid Mir, CC BY-SA 3.0, Wikimedia Commons (https://commons.wikimedia.org/wiki/File:Hamid_Mir_interviewing_Osama_bin_Laden.jpg) **D** “Cathedral” by J. Niediek is licensed under CC BY 4.0 **E** “Photo Wall” by J. Niediek is licensed under CC BY 4.0.

Fig 9 shows five examples of responses that were detected by only one of the methods. These examples illustrate that several different factors can contribute to the difference in response detection. First, small differences in cluster composition can strongly influence the numeric response score (Fig 9B, 9C and 9E). In these cases, responses are detected numerically by only one of the methods, despite relatively similar cluster composition. Second, better separation of clusters can render responses detectable that would otherwise go unnoticed (Fig 9A). Third, the fact that we require at least one spike to be fired during at least four picture presentations leads to the exclusion of some clusters that would otherwise have a low numeric response score (Fig 9D).

### Validation on whole-night recordings

**Fig 10:**
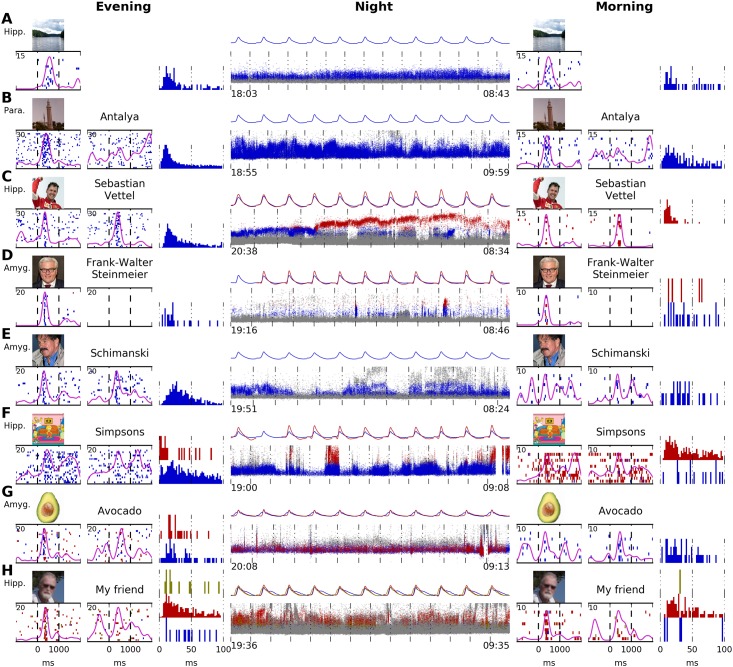
Tracking of selectively responding neurons over an entire night. **A**–**H** show data from eight patients. Continuous unit recordings started in the evening and ended the next morning. “Screening sessions” were performed at the beginning and in the end of each recording. Displayed are raster plots for one stimulus image per screening session. Inter-stimulus interval histograms for the evening and morning are displayed. In all patients but A, written names corresponding to the images were also presented. The middle column (“Night”) shows the activity of units tracked automatically during the entire recording. Each small dot marks the time point and maximal voltage of one action potential. Colors correspond to the raster plots from the screening sessions: units marked in gray do not respond to the images/written names. Units marked in blue, red, or yellow respond to the images/written names as shown in the raster plots. Mean waveforms of all responsive units are displayed for each hour recorded. **A** Stable waveform and response pattern. **B** Amplitude variations are visible. As typical for parahippocampal units, unit does not respond to the written name. **C** An amplitude shift in the responsive neuron (possibly caused by micro-movement of the electrode) results in the detection of two different units, most likely belonging to one neuron. **D** Two responsive clusters are generated. No response to the written name. **E** Stable waveform, but very weak response in the morning. **F** Solid response in the evening and morning, but with separate units. No definite conclusion about the success of tracking can be made. **G** The blue cluster generates most of the response. The red cluster also contributes to the response. Both clusters are tracked with a stable waveform. **H** Similar to G, with three responsive clusters. The red cluster generates most of the response. The blue and yellow clusters contribute to the response. All three clusters have a stable waveform. Hipp., hippocampus; Para., parahippocampal cortex; Amyg., amygdala. Stimulus pictures displayed here have been replaced by similar pictures for legal and privacy reasons. Copyright notes: **A** “Lake” by J. Niediek is licensed under CC BY 4.0 **B** “Antalya” by J. Schmidtkunz is licensed under CC BY 4.0 **C** cropped from “[…] Sebastian Vettel (Ferrari)” by Morio, CC BY-SA 4.0, Wikimedia Commons (https://commons.wikimedia.org/wiki/File:Sebastian_Vettel_2015_Malaysia_podium_2.jpg **D** cropped from “50th Munich Security Conference 2014: Vitali Klychko and Frank-Walter Steinmeier […]” by Mueller / MSC (Marc Müller), CC BY 3.0 DE, Wikimedia Commons (https://commons.wikimedia.org/wiki/File:MSC_2014_Klychko-Steinmeier3_Mueller_MSC2014.jpg) **E** “[…] Horst Schimanski […]” by H. Schrapers is licensed under CC BY-SA 2.5, Wikimedia Commons (https://commons.wikimedia.org/wiki/File:HorstSchimanski.jpg) **F** cropped from “Simpsons 20 Years” by Gabriel Shepard, CC BY-SA 3.0, DeviantArt (http://gabrielshepard.deviantart.com/art/Simpsons-20-Years-124858104) **G** “Avocado” by D. E. Bruschi is licensed under CC BY 4.0 **H** “My friend” by J. Niediek is licensed under CC BY 4.0.

Combinato was designed to work with long, possibly noisy, recordings. To test its performance on such data, we used whole-night recordings from eight epilepsy patients, for details see Section B in S1 Text. Recordings started between 18:00 and 21:00 and ended between 8:00 and 11:00 (see middle column in Fig 10 for exact times).

We used selective neuronal responses to images and written names in order to assess whether our method can track the activity of a single neuron over the course of an entire night. For this purpose we conducted a “screening session” at the beginning and end of each whole-night recording. These screening sessions differed only slightly from the experiment described in the previous section: here, eight to eleven pictures were presented to the patients. For each picture, a written representation of the picture’s content was also presented (in seven out of the eight patients). Each picture and each written name was presented ten to thirty times.

We used Combinato to extract and sort unit activity from all recordings. We then analyzed the screening sessions at the beginning and end of each recording. To illustrate our findings in a qualitative way, we selected one representative channel from each recording. Fig 10 shows the unit activity recorded in these channels from eight different patients. We chose channels carrying a selective neuronal response in both screening sessions (evening and morning), see columns “Evening” and “Morning” in Fig 10. These responses allowed to analyze whether our method splits one neuron’s activity into several different units in whole-night recordings.

The units displayed in Fig 10 exhibit several phenomena: In three cases (Fig 10A, 10B and 10E), the response to the stimulus was contained in the same cluster both in the evening and morning, and no other cluster showed any response to the same stimulus. In Fig 10A and 10B, the response raster plots from evening and morning are highly similar, whereas in Fig 10E, the response is much more pronounced in the evening than in the morning.

In the five remaining cases (Fig 10C, 10D, 10F, 10G and 10H), more than one of the generated clusters responded to the stimulus. In Fig 10C, a clear amplitude shift over the course of the night is visible in the responsive clusters. The graphical user interface can be used here to merge the two units into one continuous track. In Fig 10D, 10G and 10H, more than one stable, responsive cluster exists throughout the recording. In each of these cases, one of the tracked clusters creates the majority of the response, with small contributions by the other clusters. All clusters were successfully and independently tracked. Careful inspection of waveforms and cross-correlograms using the graphical user interface is necessary to decide whether over-clustering occurred. In the remaining case (Fig 10F), two clusters are tracked throughout the night with a stable waveform, but one cluster responds in the evening, and the other one in the morning. In this case, too, careful inspection of waveforms and cross-correlograms is necessary.

In all eight cases in Fig 10, there is a continuous background of spikes from clusters showing no response to the selected stimulus. Note that in Fig 10B and 10D there was no response to the written names of the pictures. These cells were either selective to a different semantic content of the pictures or not semantically invariant at all.

For eight more examples of units tracked throughout an entire night, see S1 Fig.

## Discussion

Spike sorting has been an important tool in electrophysiological research for decades. Existing algorithms are not optimized to be used with multi-hour datasets and do not handle noisy recordings well. We here presented a complete framework for spike sorting of multi-hour recordings under noisy conditions. Our evaluations showed that our tools outperform current spike sorting methods both on simulated data, and in the analysis of a visual stimulus presentation experiment. Furthermore, our method allows to reliably track single units in the human MTL over the course of an entire night.

### Presence of numerous, possibly sparse, neurons

Pedreira and colleagues stated that current spike sorting methods—even manually guided ones—can rarely detect more than 8–10 neurons [8]. We have shown that our automated method can reliably detect more than 10 neurons (Figs 3B and 4). Our algorithm copes with the presence of many neurons by selecting many clusters at several temperatures, and by applying SPC iteratively. Pedreira and colleagues also observe that sparsely firing neurons are particularly hard to detect with current spike sorting methods [8]. As our evaluation shows, our method is capable of correctly detecting sparse neurons, e.g. units C10 and C11 in Fig 3C, consisting of 220 and 73 spikes, respectively (2.21% and 0.73% of the 9942 spikes that were the input to clustering).

### Large numbers of clusters per channel

In many cases, our automated method generated more units than manual operators of WaveClus (Fig 8D). This could be due to several reasons. First, our post-sorting artifact rejection sometimes missed artifact clusters, e.g. unit D 4 in Fig 7D. Such missed artifact clusters artificially increase the unit count. Since no ground truth is available for artifact clusters, the accuracy of our post-sorting artifact rejection is difficult to estimate.

Second, there are cases where manual operators of WaveClus failed to separate two or more true units. An example is provided in Fig 7D through 7F: the raster plots (Fig 7F) show that our method correctly separated units D 1 and D 2, while operators of WaveClus generated the under-clustered unit E 1.

Third, as Fig 8E indicates, over-clustering occurred more frequently in our method than with manual operators of WaveClus. To avoid any bias introduced by over-clustering, we counted no more than one responding cluster per stimulus and channel, so that over-clustering could not artificially increase the number of responses. With and without this correction, our method detected more neuronal responses than manual operators of WaveClus. When the goal is to maximize the number of detected responses, a potentially increased likelihood of over-clustering is justified. However, by modifying Combinato’s parameters, researchers can systematically shift the balance between over- and under-clustering according to the demands of the respective scientific question, an option (to our knowledge) not available in other spike sorting methods.

As summarized in [8], theoretical considerations predict higher numbers of neurons per recording channel than typically observed with current spike sorting techniques. Thus our result might represent unit counts more realistically than other methods.

### Properties of block-wise sorting

We segmented spikes into blocks for spike sorting. This has various advantages over spike sorting all spikes at once: First, periods of signal contamination are often confined to short segments of the recording, and thereby affect only a small number of blocks. The remaining, uncontaminated blocks are spike-sorted independently, without the detrimental effects of large numbers of non-neural artifacts.

Second, especially in multi-hour sleep recordings, some units may be active only during short parts of the recording. In a block-wise approach, these units have high chances of being detected in the corresponding blocks, but might be overlooked if spikes from the entire recording were sorted in one step.

Third, the computational time of spike sorting algorithms typically scales super-linearly with the number of spikes sorted. A block-wise approach not only avoids these super-linear computational costs, but also enables us to use a parallelized implementation for the sorting of different blocks.

We used a fixed number of spikes per block (20 000 by default). Other ways of defining blocks are conceivable, e.g. a fixed amount of recording time for each block. There are two possible problems with a time-based definition of blocks: First, clinical recordings often suffer from short periods of signal contamination, during which a large number of artifactual spikes is generated. In a time-based approach, short periods of signal contamination thus pollute blocks of otherwise uncontaminated recording. In our approach, if large numbers of artifactual spikes are generated, these are confined to blocks that correspond to short amounts of recording time.

Second, in a time-based approach, researchers would necessarily have to adapt the block length to the firing rates of the neurons recorded. Without such an adaptation, there would be the risk of accumulating too few or too many spikes for successful spike sorting in each block. However, even though this adaptation could be performed automatically, it would introduce another algorithmic step without obvious benefits.

Several alternative approaches to the problem of sorting hundreds of thousands of spikes are conceivable. One idea would be to generate clusters from a small random subset of all spikes and use these clusters as templates in a template matching procedure. A possible problem with this approach is that sparse units could be missed if no corresponding template was generated. Furthermore, even short periods of signal contamination can lead to the presence of large amounts of non-neural artifacts, which could in turn compromise the template matching procedure if no templates for the artifacts exist. Another idea is implemented in spike sorting tools that use template matching as the main principle for clustering [7, 11]. While these methods have the advantage of working online, they continuously have to solve the problem of when to open a new cluster, based on single spikes. This decision problem might become harder in the presence of non-neural artifacts. Further studies are necessary to determine how such methods perform in comparison to our framework.

### Applicability to neuroscientific studies

The four validation schemes used in this study show that our framework is ready for use in a neuroscientific study. Fig 7D demonstrates our method’s ability to spike-sort highly contaminated recordings.

Fig 8G shows response counts for each region we recorded from. Because each response is defined as a pair of a stimulus and a neuronal unit, individual units can contribute more than once to the counts. Several studies report the fraction of units that respond to at least one stimulus (14% of all units in the MTL [15]; 11% of all units in the MTL [22]; 9–16% of all units in individual MTL subregions [17]). These studies also report responses by one neuron to more than one stimulus, either by example [22] or as a summary statistic: in one study, the average percentage of stimuli eliciting a response in a responsive neuron was 4.7% in the parahippocampal cortex, 1.7% in the entorhinal cortex and hippocampus, and 2.4% in the amygdala [17].

An exhaustive analysis of response counts is beyond the scope of the present work. Nevertheless, we report here the response statistics for the Combinato sorting: 11.9–26.7% of all units responded to at least one stimulus, depending on MTL subregions. The average percentage of stimuli eliciting a response in a responsive neuron was 3.7% in the parahippocampal cortex, 1.5% in the entorhinal cortex, 2.3% in the hippocampus, and 2.1% in the amygdala.

The average percentages of response-eliciting stimuli per responsive neuron we observed are in good agreement with the findings of [17]. As discussed in [17], a likely reason for the higher average number of response-eliciting stimuli in the parahippocampal cortex with respect to the other regions is that parahippocampal units respond less selectively to the stimulus material used here.

Our evaluation of whole-night recordings demonstrates the feasibility of tracking responsive units in the human MTL over the course of an entire night. This might provide the means to address some important neuroscientific questions for the first time. Apart from mechanisms related to memory consolidation, processes underlying the generation of epileptic seizures are a relevant topic of research [23]. A question of particular interest would be how firing patterns of single neurons change in the hours before an epileptic seizure [24].

Micro-electrode recordings from the human brain are a novel and still developing technique. Our method can be used to evaluate the stability of multi-hour recordings in order to optimize recording procedures and to identify potential problems and pitfalls. Suitable methods to assess recording stability have been proposed by several authors [25, 26]. Another interesting question concerns the activity of visually responsive neurons during sleep. Bondar and colleagues [27] addressed questions regarding the response stability of such neurons in the infero-temporal cortex of rhesus monkeys, by comparing responses across different recording sessions. However, a similar study with human subjects has not been published, and the activity patterns of such neurons during sleep are yet unknown.

### Future directions

We concatenated existing simulated datasets to evaluate our algorithms on multi-hour data where ground truth is available. Despite the fact that we increased the difficulty of the spike sorting task by adding drift and noise, our algorithms correctly identified 74.6% of the simulated units. For further quantitative evaluations, simulation algorithms as in [28, 29] could be used to create multi-hour datasets with ground truth available.

Many spike sorting algorithms employ template matching at some point. Template matching can be performed in many different ways. Our template matching algorithm is based on the Euclidean distance in waveform space. Rutishauser and colleagues use distances based on the Euclidean distance, with the option to use pre-whitened waveforms [7]. Friedman and colleagues use a more complex matching method (“rebuilding from cores”). Integrating such template matching algorithms into our tools could lead to further improvement [10].

The trade-off between over- and under-clustering deserves further investigation. As we have shown, our methods make it possible to automatically sample a wide range of settings, which directly influence unit counts. Depending on the specific research question at hand, different points along this parameter range will prove optimal.

In contrast to human recordings, animal electrophysiologists have long been using stereotrodes, tetrodes, and multitrodes, which increase both clustering quality and unit yield [25, 30]. As of yet, our software has been tested only with single-wire electrodes. An adaptation to multi-wire electrodes would require only small changes in the code and would certainly broaden the scope of our software.

## Ethics Statement

This study was approved by the Medical Institutional Review Board of the University of Bonn Medical Center (approval number 095/10, implantation of micro-electrodes; approval number 242/11, whole-night recordings and data analysis). All patients gave informed written consent.

## Supporting Information

S1 Fig Tracking of selectively responding neurons over an entire night. (PDF)

S1 Text A Spike extraction and storage. B Recordings from epilepsy patients.
(PDF)

S2 Text Installation instructions for Combinato. (PDF)

S3 Text User tutorial for Combinato. (PDF)
