# Supplementary material for: Reliable Analysis of Single-Unit Recordings from the Human Brain under Noisy Conditions: Tracking Neurons over Hours
Source: PLoS One. 2016 Dec 8;11(12):e0166598. doi: 10.1371/journal.pone.0166598 (PMC5145161; doi:10.1371/journal.pone.0166598)
Supplement: S1 Fig — (PDF) [file pone.0166598.s001.pdf]

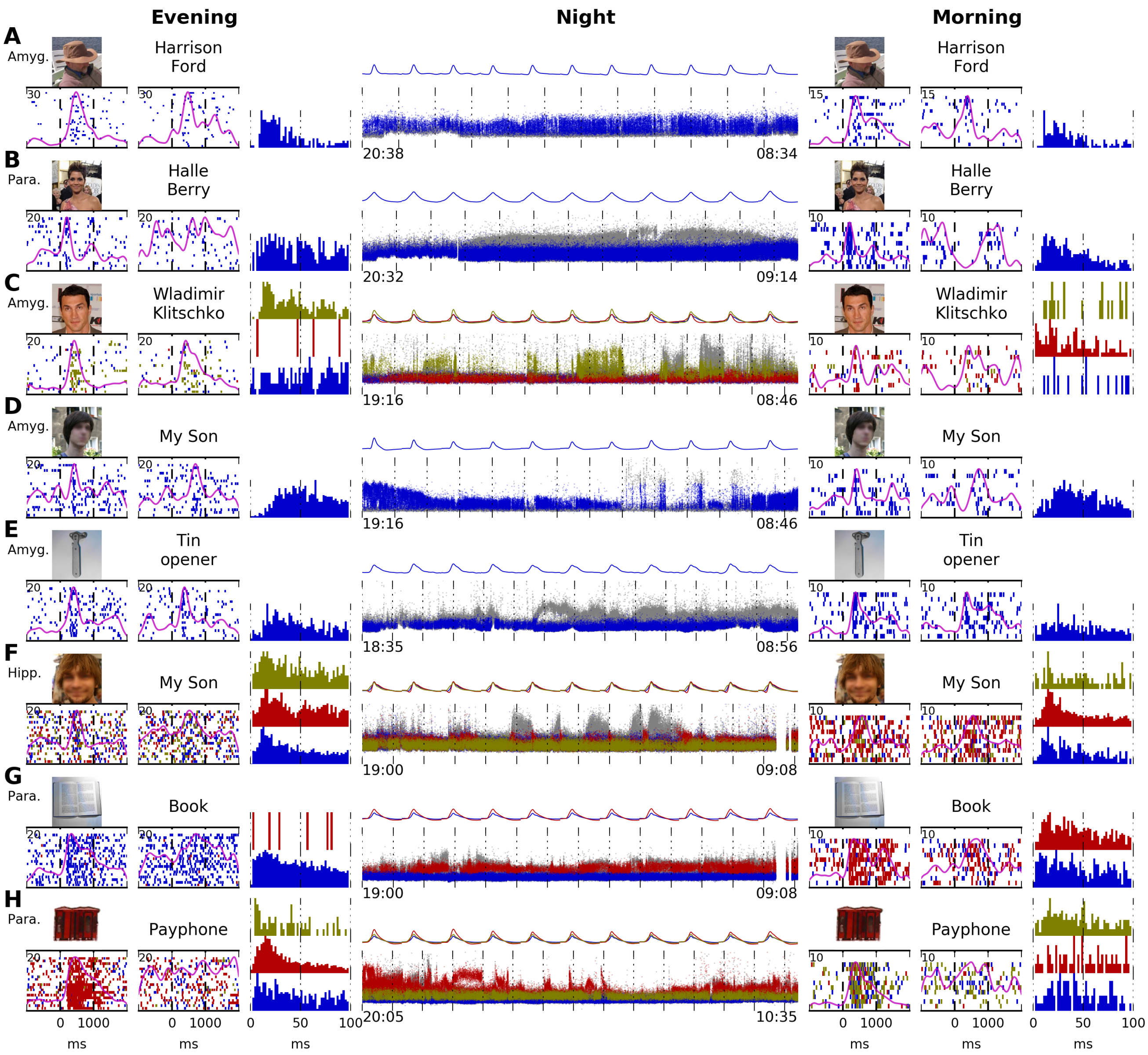

S1 Fig. Tracking of selectively responding neurons over an entire night. A–H show continuous unit recordings starting in the evening and ending the next morning. “Screening sessions” were performed at the beginning and in the end of each recording. Displayed are raster plots for one stimulus image per screening session. In all sessions, written names corresponding to the images were also presented. Inter-stimulus interval histograms for the evening and morning are displayed. The middle column (“Night”) shows the activity of units tracked automatically during the entire recording. Each small dot marks the time point and maximal voltage of one action potential. Colors correspond to the raster plots from the screening sessions: units marked in gray do not respond to the images/written names. Units marked in blue, red, or yellow respond to the images/written names as shown in the raster plots. Mean waveforms of all responsive units are displayed for each hour recorded. **A**, **B**, **D**, **E** One responsive unit was continuously tracked throughout each recording. **C**, **F**, **G**, **H** Two, resp. three responsive units were continuously tracked throughout each recording. However, contributions to visual responses of each unit sometimes differ between evening morning and evening. As is often the case in the parahippocampal cortex, units do not respond to written names. Hipp., hippocampus; Para., parahippocampal cortex; Amyg., amygdala. Stimulus pictures displayed here have been replaced by similar pictures and/or modified for legal and privacy reasons. Copyright notes: **A** “Indiana Jones” by J. Niediek is licensed under CC BY 4.0 **B** cropped from “Halle Berry at the Golden Globes 2013” by Jenn Deering Davis, CC BY 2.0, Wikimedia Commons (`https://commons.wikimedia.org/wiki/File:Halle_Berry_2013.jpg`) **C** cropped from “Wladimir Klitschko at a press conference in Germany” by Michael Schilling, CC BY-SA 3.0, Wikimedia Commons (`https://commons.wikimedia.org/wiki/File:Wladimir-Klitschko.jpg`) **D** “My Son” by J. Niediek is licensed under CC BY 4.0 (face blurred) **E** “Tin Opener” by J. Niediek is licensed under CC BY 4.0 **F** “My Son” by J. Niediek is licensed under CC BY 4.0 (face blurred) **G** “Book” by J. Niediek is licensed under CC BY 4.0 **H** “Payphone” by J. Schmidtkunz is licensed under CC BY 4.0.
