# Supplementary material for: Reliable Analysis of Single-Unit Recordings from the Human Brain under Noisy Conditions: Tracking Neurons over Hours
Source: PLoS One. 2016 Dec 8;11(12):e0166598. doi: 10.1371/journal.pone.0166598 (PMC5145161; doi:10.1371/journal.pone.0166598)
Supplement: S1 Text — A Spike extraction and storage. B Recordings from epilepsy patients. (PDF) [file pone.0166598.s002.pdf]

A Spike extraction and storage

Spike extraction is independent from any specific sorting algorithm. The principles of our spike extraction procedures resemble WaveClus [1]. However, our implementation is optimized both for computational speed and for large datasets.

Data are bandpass filtered between 300 Hz and 1000 Hz (second order elliptic filter), and a threshold is determined as $\theta := \frac{5}{0.6745} \operatorname{median}(\operatorname{abs}(\mathbf{x}))$, where $\mathbf{x}$ is the filtered signal. The threshold is then used to determine the timepoints of events for extraction. Spikes can occur as both positive and negative voltage deflections. Because event extraction is computationally inexpensive in comparison to data reading and filtering, our software extracts by default *both* positive voltage deflections above θ *and* negative voltage deflections below $-\theta$. These positive and negative voltage deflections are stored as two different arrays for later sorting. For each threshold-crossing event, 64 sampling points (corresponding to approximately 2 ms at the sampling rates used here) are extracted from the signal after bandpass-filtering between 300 Hz and 3000 Hz (second order elliptic filter), and aligned to maximum after upsampling using cubic spline interpolation.

We implemented the extraction algorithm in the following, computationally efficient, way: Instead of reading and processing data in an alternating manner, one process continuously reads data segments from disk into memory, while *simultaneously* many processes (typically, one per core of the computer) run in parallel to filter the signal and to extract and store spikes. In this way, our implementation maximizes input bandwidth utilization, thereby minimizing the time processors spend idle [2].

The extracted spike waveforms are stored as an array, along with one time stamp per spike. We use the standardized HDF5 data format, which allows for efficient processing of very long recordings.

B Recordings from epilepsy patients

We used existing datasets from ongoing and completed studies in our laboratory to evaluate the performance of our algorithms. The following summarizes the data acquisition procedure used in these studies.

Ten epilepsy patients (four female, age range 21–48, mean age 35.2) were implanted with intracerebral depth electrodes to localize the epileptic focus. A typical implantation scheme consisted of one to three depth electrodes in the hippocampus bilaterally, and one depth electrode in each of the following MTL subregions bilaterally: amygdala, entorhinal cortex, and parahippocampal cortex. Electrode locations were defined exclusively by clinical criteria. To confirm correct electrode placement, computer tomography co-registered to pre-operative MRI as well as post-operative MRI were used in each patient.

Each depth electrode contained a bundle of nine micro-electrodes protruding from its tip (AdTech, Racine, WI). Each bundle consisted of eight high-impedance recording electrodes and one low-impedance reference electrode. The high-impedance electrodes were used to record single- and multi-unit activity. As a

reference electrode, we either selected one high-impedance micro-electrode in each electrode bundle as a local reference (bipolar recordings, four patients), or one global low-impedance electrode (referential recordings, six patients). The recording mode (bipolar or referential) was chosen depending on technical and clinical demands.

The micro-electrode signals were recorded on a *Digital Lynx SX* (four patients) or *ATLAS* system (six patients; Neuralynx, Bozeman, MT) at an effective recording bandwidth of 0.1 Hz to 9000 Hz. Recordings were digitized at 32 000 Hz or 32 768 Hz depending on technical and clinical demands, and transferred to network-attached storage units for further analysis.

For the present study, two types of experiments were performed: Picture presentation experiments and whole-night recordings. For four patients, both types of recordings were analyzed here. For two patients, only the picture presentation experiment was analyzed here. For four patients, only the whole-night recordings were analyzed here.

This study was approved by the Medical Institutional Review Board of the University of Bonn Medical Center (approval number 095/10, implantation of micro-electrodes; approval number 242/11, whole-night recordings and data analysis). All patients gave informed written consent.

References

1. Quian Quiroga R, Nadasdy Z, Ben-Shaul Y. Unsupervised Spike Detection and Sorting with Wavelets and Superparamagnetic Clustering. Neural Comput. 2004;16(8):1661–1687. doi:10.1162/089976604774201631.

2. Alted F. Why Modern CPUs Are Starving and What Can Be Done about It. Comput Sci Eng. 2010;12(2):68–71. doi:10.1109/MCSE.2010.51.
