# Supplementary material for: Reliable Analysis of Single-Unit Recordings from the Human Brain under Noisy Conditions: Tracking Neurons over Hours
Source: PLoS One. 2016 Dec 8;11(12):e0166598. doi: 10.1371/journal.pone.0166598 (PMC5145161; doi:10.1371/journal.pone.0166598)
Supplement: S2 Text — (PDF) [file pone.0166598.s003.pdf]

Installation Instructions for Combinato

Johannes Niediek

Contents

General Remark **1**

Combinato Installation Instructions for Linux **2**
1. Getting the code 2
2. Installing dependencies 2
3. Setting up the environment 2
   3.1 Choosing the right clustering backend 2
   3.2 Setting Paths 2
4. Testing the Installation 3

Combinato Installation Instructions for Windows **4**
1. General Remark 4
2. Installing Python 4
3. Getting the Combinato code 4
4. Setting Paths 4
5. Testing the Installation 5
6. Using the Combinato Scripts 5

Combinato Installation Instructions for OS X **6**
1. Installing Python 6
2. Getting the Combinato code 6
3. Setting Paths 6
4. Testing the Installation 7

General Remark

The documentation of Combinato, including the installation instructions, is maintained as a Wiki at https://github.com/jniediek/combinato/wiki. This PDF file was created from the Wiki automatically by Pandoc (http://pandoc.org).

In case of any questions regarding the installation or usage of Combinato, feel free to contact Johannes Niediek (`jonied@posteo.de`).

Combinato Installation Instructions for Linux

1. Getting the code

If you use git, clone the repository:

`git clone https://github.com/jniediek/combinato.git`

If you do not use git, download Combinato from https://github.com/jniediek/combinato/archive/master.zip.

2. Installing dependencies

It is possible to skip this step and come back in case of dependcy problems.

Combinato depends on `scipy`, `pywt`, `matplotlib`, `pytables`, and `pyqt`. Most modern Linux distributions have these packages installed by default. If you miss any of these packages, use your distribution's package manager to install them.

For example, in Ubuntu and Debian

`sudo apt-get install python-scipy python-matplotlib python-pywt python-tables python-qt4`

installs all dependencies.

3. Setting up the environment

3.1 Choosing the right clustering backend

Combinato uses a compiled executable file as backend for clustering. To automatically select the correct file for your operating system, navigate your shell to the Combinato repository and run

`python setup_options.py`.

3.2 Setting Paths

Combinato is organized as a collection of executable scripts. These are the files named `css-*` in the repository's main folder. To be able to execute these scripts, add the repository's folder to your `$PATH` as shown below.

For some of the tools in the subdirectories `signalviewer` and `tools`, it is necessary to add the repository's folder to `$PYTHONPATH` as well.

For example, my Combinato repository is in

```
/home/johannes/combinato
```

and my shell's configuration (`/home/johannes/.bashrc`) contains

```
PATH=$PATH:/home/johannes/combinato
PYTHONPATH=$PYTHONPATH:/home/johannes/combinato
export PATH PYTHONPATH
```

4. Testing the Installation

1. Open a new shell
2. Navigate to the Combinato repository
3. Enter `python tools/test_installation.py`

In case everything worked, you will see the following output:

```
Found Combinato
Found SPC binary
Your version of pytables is 3.2.2
Combinato clustering setup: no problems detected.
Found display
Found 'montage', plotting continuous data possible.
```

Combinato Installation Instructions for Windows

1. General Remark

Combinato works well on Windows, but it is primarily developed for Linux. Combinato is organized as a set of command line tools, which is optimal for remote servers and shell scripting, but might be cumbersome on Windows computers.

So please consider using Combinato on Linux (or OS X). If you decide to use Windows, here are the instructions.

2. Installing Python

Any Python distribution should work. We use Combinato with Anaconda for Windows by Continuum. For now, please use the Python 2.7 version.

You can download Anaconda from https://www.continuum.io/downloads.

Anaconda contains all packages that Combinato depends on (`scipy`, `matplotlib`, `pytables`, `pywt`, `pyqt`).

If you are completely new to Python, you can now run the Anaconda "Test Drive" at http://conda.pydata.org/docs/test-drive.html.

3. Getting the Combinato code

If you use git, clone the repository:

`git clone https://github.com/jniediek/combinato.git`

If you do not use git, download Combinato from https://github.com/jniediek/combinato/archive/master.zip.

Save the repository to a convenient place, for example

`C:\Users\YourUsername\Anaconda\Lib\site-packages`.

4. Setting Paths

Combinato uses an executable file as backend for clustering. To automatically select the correct file for your operating system, navigate your shell to the Combinato repository and run

`python setup_options.py`.

5. Testing the Installation

1. Open a new shell
2. Navigate to the Combinato repository
3. Enter `python tools/test_installation.py`

In case everything worked, you will see the following output:

```
Found Combinato
Found SPC binary
Your version of pytables is 3.2.2
Combinato clustering setup: no problems detected.
Found display
Found 'montage', plotting continuous data possible.
```

If you see `Plotting continuous data will not work`, don't worry. This feature requires ImageMagick, but it is not crucial.

6. Using the Combinato Scripts

In Windows, you cannot simply run the scripts named `css-*`, but you have to prepend `python` to each command. For example, to run `css-simple-clustering`, enter

`python css-simple-clustering`,

or, if the current directory of your command prompt is different from the Combinato repository (this will almost always be the case!),

`python C:\Path\To\Combinato\css-simple-clustering`.

This is cumbersome, and this problem does not exist with Linux and OS X. See the FAQ to learn why Combinato is made this way.

Combinato Installation Instructions for OS X

1. Installing Python

Any Python distribution should work. We use Combinato with Anaconda for OS X by Continuum. For now, please use the Python 2.7 version.

You can download Anaconda from https://www.continuum.io/downloads.

Anaconda contains all packages that Combinato depends on (`scipy`, `matplotlib`, `pytables`, `pywt`, `pyqt`).

If you are completely new to Python, you can now run the Anaconda "Test Drive" at http://conda.pydata.org/docs/test-drive.html.

2. Getting the Combinato code

If you use git, clone the repository:

```
git clone https://github.com/jniediek/combinato.git
```

If you do not use git, download Combinato from https://github.com/jniediek/combinato/archive/master.zip.

Save the repository to a convenient place, for example

`/Applications/anaconda/lib/python2.7/site-packages`.

3. Setting Paths

Combinato uses an executable file as backend for clustering. To automatically select the correct file for your operating system, navigate your shell to the Combinato repository and run

`python setup_options.py`.

Combinato is organized as a collection of executable scripts. These are the files named `css-*` in the repository's main folder. To be able to execute these scripts, add the repository's folder to your `$PATH`: The path is set in the file `.bash_profile` in your user directory. For example, this file should contain a line like

`export PATH=$PATH:/Applications/anaconda/lib/python2.7/site-packages/combinato`.

For some of the tools in the subdirectories `signalviewer` and `tools`, it is necessary to add the repository's folder to `$PYTHONPATH` as well.

4. Testing the Installation

1. Open a new shell
2. Navigate to the Combinato repository
3. Enter `python tools/test_installation.py`

In case everything worked, you will see the following output:

```
Found Combinato
Found SPC binary
Your version of pytables is 3.2.2
Combinato clustering setup: no problems detected.
Found display
Found 'montage', plotting continuous data possible.
```

If you see `Plotting continuous data will not work`, don't worry. This feature requires ImageMagick, but it is not crucial.
