# Supplementary material for: Reliable Analysis of Single-Unit Recordings from the Human Brain under Noisy Conditions: Tracking Neurons over Hours
Source: PLoS One. 2016 Dec 8;11(12):e0166598. doi: 10.1371/journal.pone.0166598 (PMC5145161; doi:10.1371/journal.pone.0166598)
Supplement: S3 Text — (PDF) [file pone.0166598.s004.pdf]

Combinato Tutorial

Johannes Niediek

Contents

General remark **1**

Tutorial Part I **2**
1. Download sample data 2
2. Extract spikes 2
3. Cluster the spikes 2
4. Check the results 2
   4.1 Using overview plots 3
   4.2 Using the graphical user interface 4

Tutorial Part II **6**
1. What's the problem? 6
2. Fix the problem by parameter tuning 7
3. Manual optimization 7

Tutorial Part III **9**
1. Download the data 9
2. Extract spikes and mask artifacts 9
3. Cluster the data 11
4. Check the results 12
   4.1 Using overview plots 12
   4.2 Using the graphical user interface 12
5. Outlook 15
6. Working with raster plots 15

General remark

The Combinato documentation, including this tutorial, is maintained as a Wiki at https://github.com/jniediek/combinato/wiki. This PDF file was automatically created from the Wiki by Pandoc (http://pandoc.org).

Feel free to contact Johannes Niediek (`jonied@posteo.de`) in case of any questions regarding Combinato.

Tutorial Part I

1. Download sample data

In this part of the tutorial, we will work with synthetic (simulated) data. We will work with the file

http://bioweb.me/CPGJNM2012-dataset/simulation_5.mat.

Download the file.

For the curious

The simulated data was produced by Carlos Pedreira and colleagues. Details can be found in:

Pedreira, C., Martinez, J., Ison, M. J., Quian Quiroga, R.: "How many neurons can we see with current spike sorting algorithms?" Journal of Neuroscience Methods 211 (1), 2012. doi:10.1016/j.jneumeth.2012.07.010 http://www.sciencedirect.com/science/article/pii/S0165027012002749.

2. Extract spikes

After downloading the simulated data, move the command prompt to the folder where you stored it and enter

`css-extract --matfile simulation_5.mat`.

There is now a folder `simulation_5`, containing one file `data_simulation_5.h5`.

If you would like to use your own Matlab file, store the data in a variable `data` and the sampling rate in a variable `sr`.

3. Cluster the spikes

In this tutorial we try to keep things simple. So just enter

`css-simple-clustering --datafile simulation_5/data_simulation_5.h5`.

After a few seconds, the folder `simulation_5` contains the sorted data.

4. Check the results

Now we would like to see the output of the clustering procedure. There are two main ways for visualization: overview plots and the graphical user interface.

4.1 Using overview plots

Enter

`css-plot-sorted --label sort_pos_simple`.

There is now a folder `overview`, containing just one plot:

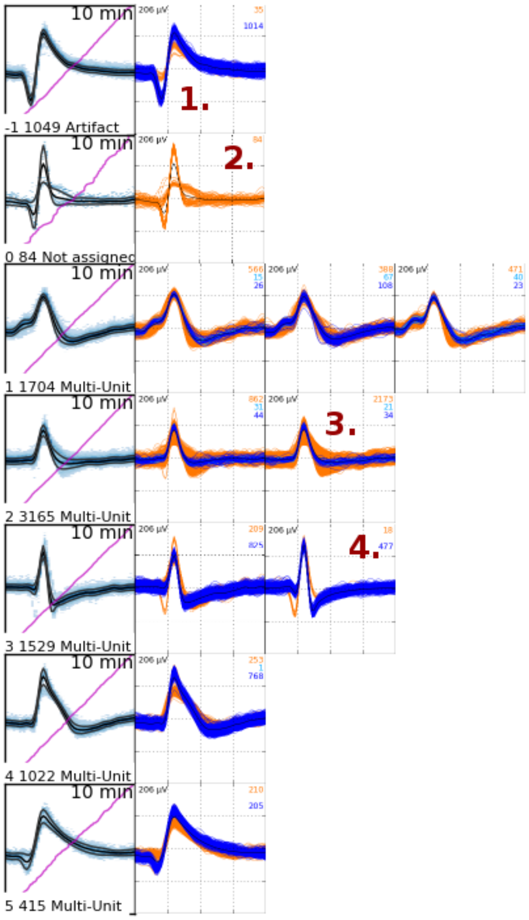

Figure 1: Clusters from Simulation 5

The red numbers 1. through 4. point at a few problems with the clustering that we are going to solve in the next part of the tutorial.

4.2 Using the graphical user interface

Enter `css-gui` and click on File, Open. You will see a list containing just one file, our Simulation 5. Click on OK.

You will see this (or a similar) screen:

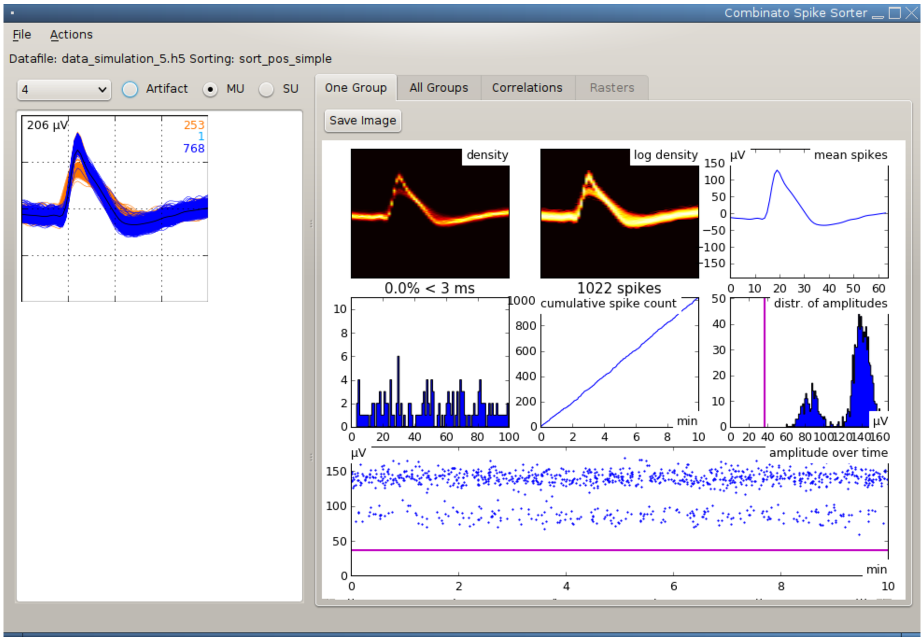

Figure 2: Simulation 5 Cluster 4 in the GUI

Go through the individual cluster groups by using the pull down menu. The plots on the right hand side update automatically. In our screenshot you can see that the unit displayed is "under-clustered": it should be split apart further. We are going to solve this problem in the next part of the tutorial.

Now, click on the "All Groups" tab. You will see this (or a similar) screen:

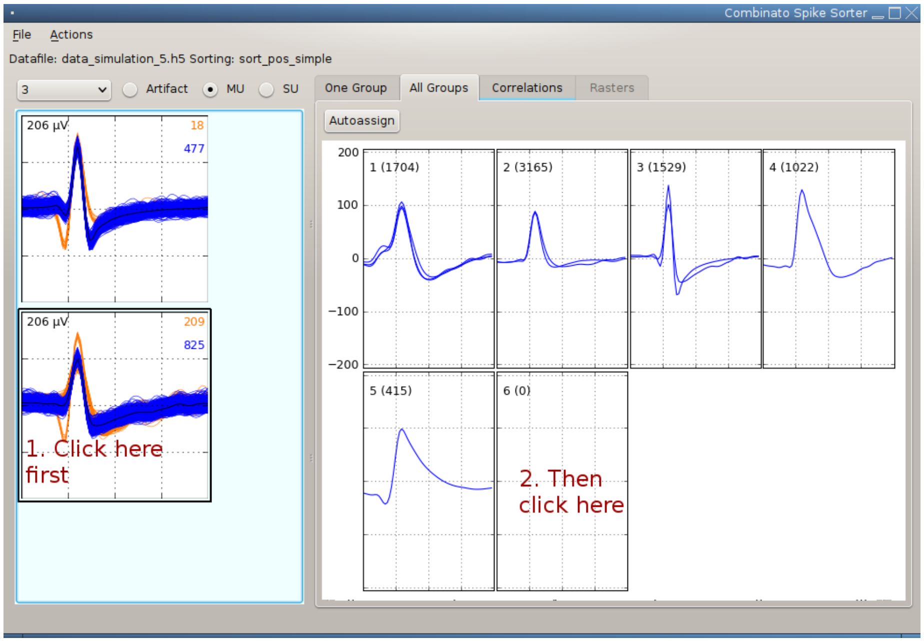

Here you see an overview of all cluster groups contained in Simulation 5. In some cases, clusters are grouped together in the wrong way. To solve this:

- First create a new group by clicking Actions, New Group.
- Then click on one of the clusters you would like to move to the new group (red 1. in the screenshot)
- The last step is to click into the new, empty group (red 2. in the screenshot).

You can save your modification by clicking File, Save.

Tutorial Part II

1. What's the problem?

We saw that Combinato created the following clustering result:

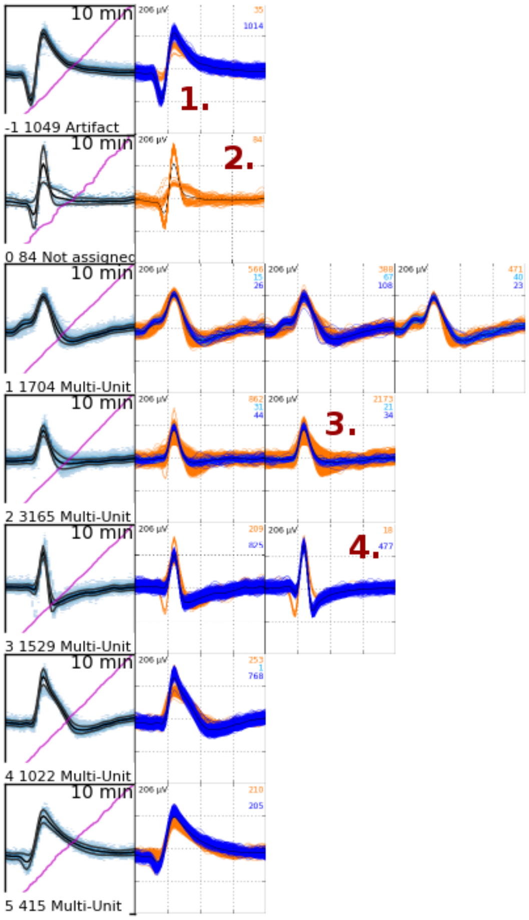

Figure 3: Clustering result of Simulation 5

The problems are (the numbers refer to the red numbers in the plot):

1. A cluster was wrongly designated an artifact.
2. Some spikes were not assigned to any cluster.
3. This is a multi-unit that should be further split apart.
4. There are some spikes in this unit that should not be part of it.

2. Fix the problem by parameter tuning

Create a file called `local_options.py` in the same folder that contains the `simulation_5` folder. The content of the file is the following:

```
options = {'MaxClustersPerTemp': 7,
           'RecursiveDepth': 2,
           'MinInputSizeRecluster': 1000,
           'MaxDistMatchGrouping': 1.6,
           'MarkArtifactClasses': False,
           'RecheckArtifacts': False}
```

Then re-run the clustering procedure. At this point, you should use a different *label*. Labels are names under which the clustering results are stored. By using different labels, you can save different clustering results from the same data and compare them later. So just enter

`css-simple-clustering --datafile simulation_5/data_simulation_5.h5 --label optimized`.

When the process is finished, enter

`css-plot-sorted --label sort_pos_optimized`.

(The prefix `sort_pos_` is automatically prepended to the label).

The sorting results are much better now:

As you can see, with the optimized options, Combinato generated 10 units. Each unit is displayed as a density plot along with its cumulative spike count (see the red frame for an example). Just next to the density plots, there is a list of all subclusters the unit consists of.

1. Unit 1 consists of 8 subclusters. Probably the 5th and 7th subclusters should be made a different unit.
2. Unit 3 consists of 2 subclusters. These are very different and should be split into two units using `css-gui`.
3. Unit 7 consists of 2 subclusters. The first of these could be split further apart.

3. Manual optimization

As explained in Part I, use `css-gui` to further split apart under-clustered units. You can also set units to *Single Unit* in `css-gui` (all units are considered multi-units by default):

If you then save your modifications and re-plot the results (`css-plot-sorted --label sort_pos_optimized`), the result will be this:

This is a rather nice result. Congratulations!

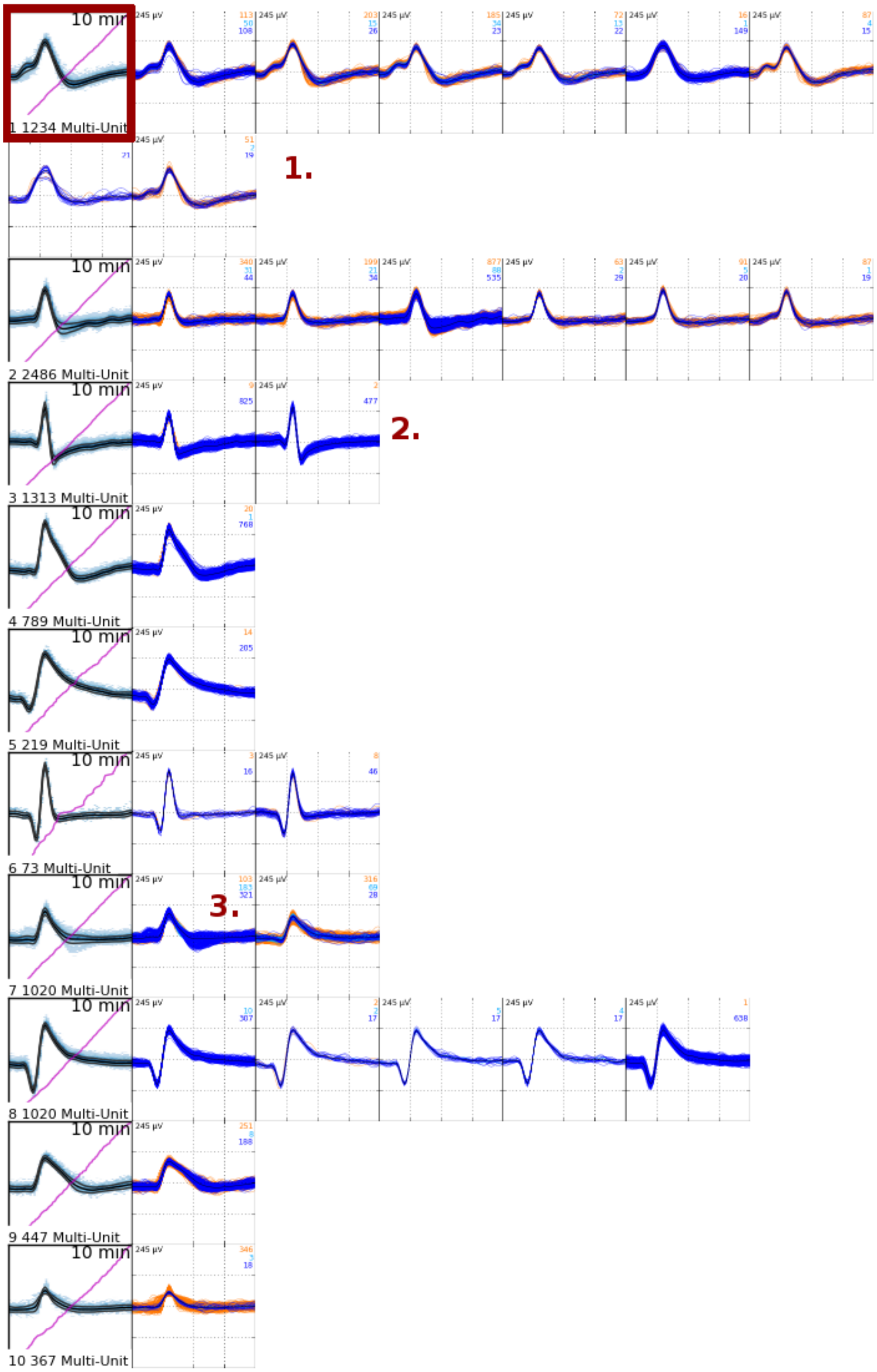

Figure 4: Optimized clustering results from Simulation 5

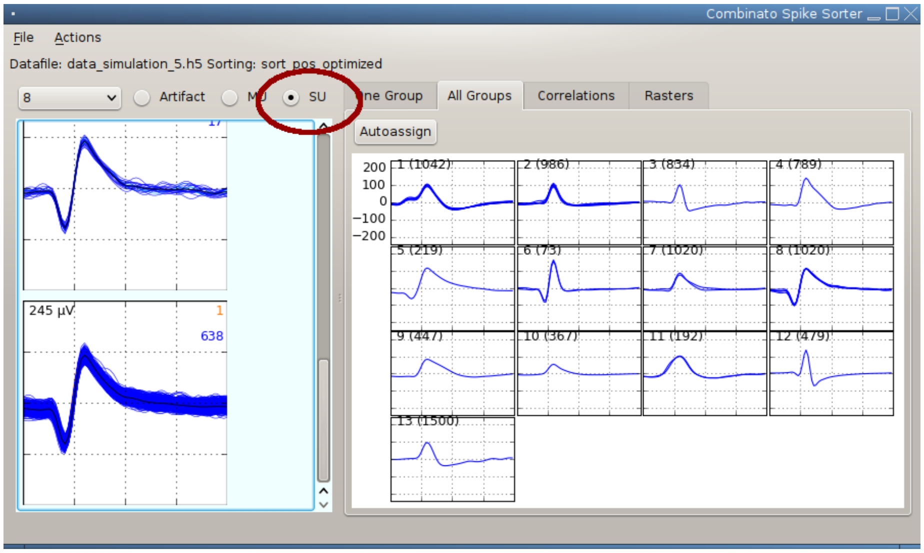

Figure 5: Setting units to single unit

Tutorial Part III

In this part of the tutorial we will finally work with real data recorded from the right hippocampus.

1. Download the data

Download the file `CSC67.ncs` from

https://uni-bonn.sciebo.de/index.php/s/K1NLbTjVmO1Hx19.

The password is `combi_data`.

2. Extract spikes and mask artifacts

First make sure to delete the `local_options.py` and `local_options.pyc` files if they are still around them from a previous part of the tutorial.

Similar to before, run

```
css-extract --files CSC67.ncs
```

to extract spikes. Then run

```
css-mask-artifacts --datafile CSC67/data_CSC67.h5
```

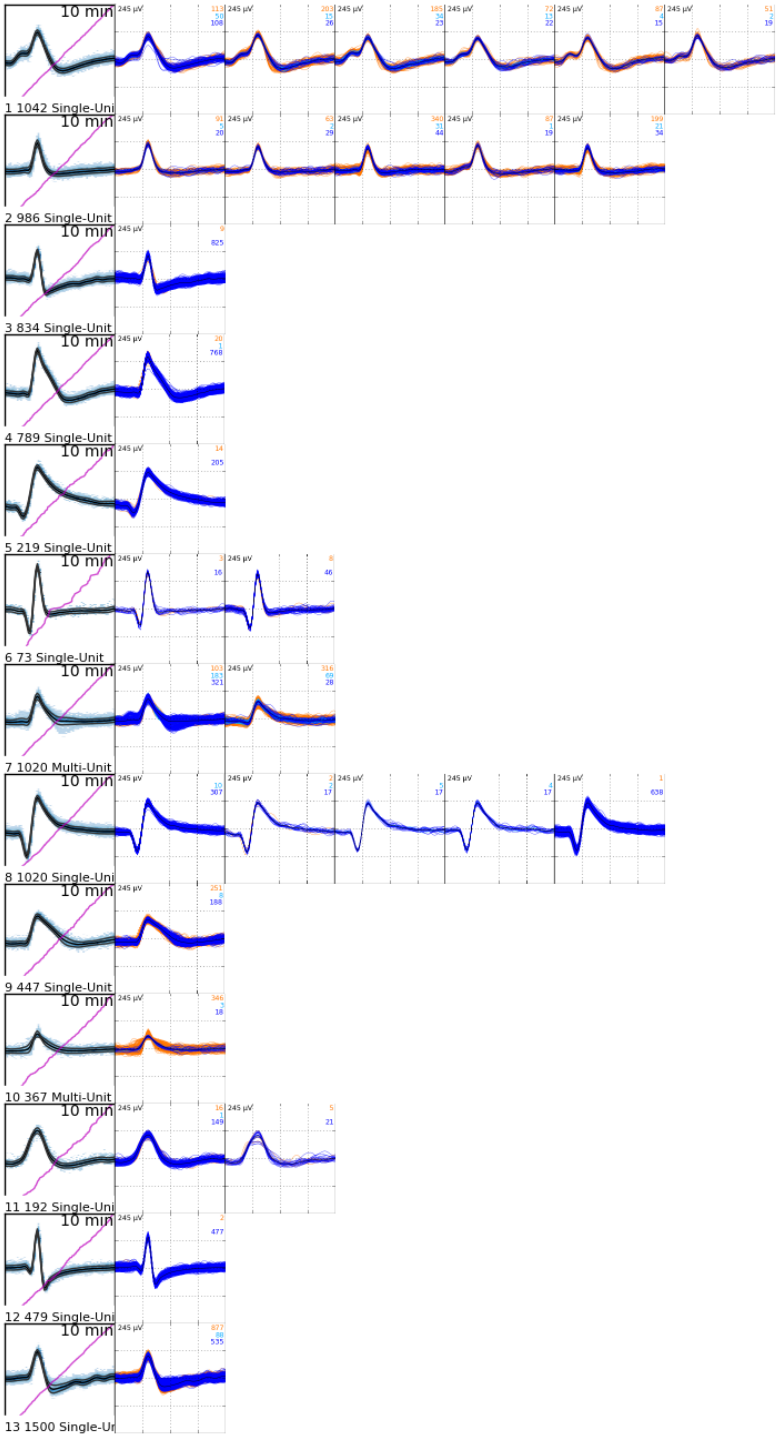

Figure 6: Manually optimized clustering results of Simulation 5

to mask artifacts before spike sorting.

For the curious

- Run `css-plot-rawsignal` to create an overview plot of the raw signal.
- Run `css-plot-extracted --datafile CSC67/data_CSC67.h5` to create an overview plot of the extracted spikes before sorting.

Both plots are saved in the `overview` folder:

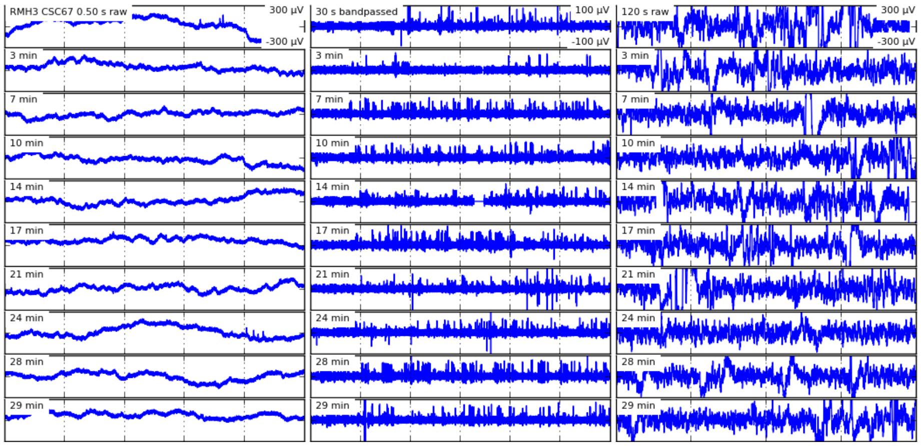

Figure 7: Plot of raw signal

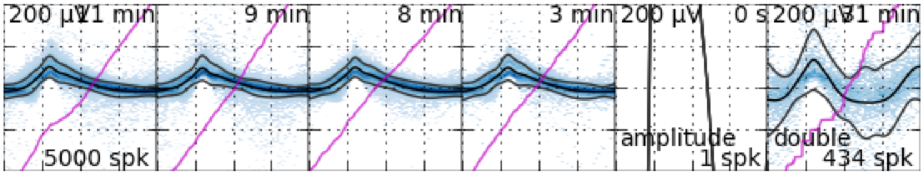

Figure 8: Plot of extracted spikes

You can use the program `css-overview-gui` to visualize summary statistics of all channels, and to quickly display the overview plots.

3. Cluster the data

Just as before, run

```
css-simple-clustering --datafile CSC67/data_CSC67.h5
```

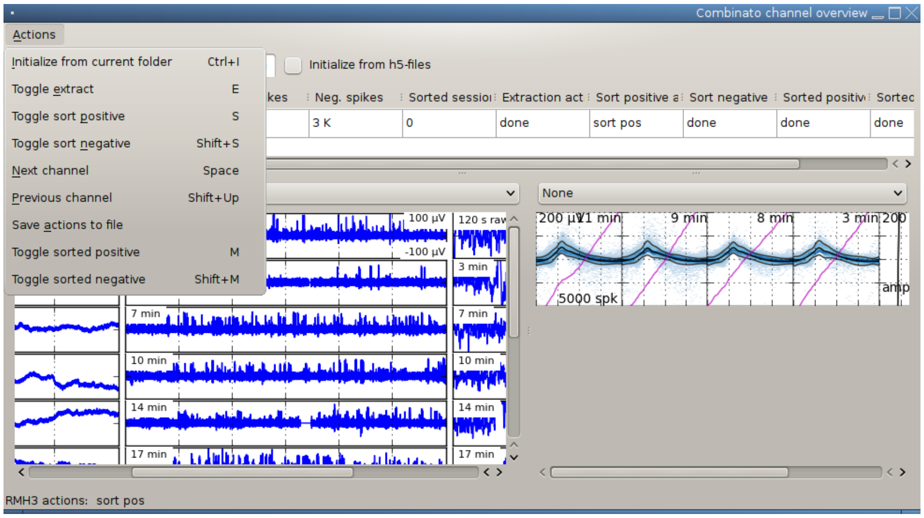

Figure 9: Screenshot of overview-gui

4. Check the results

4.1 Using overview plots

Run

`css-plot-sorted --datafile CSC67/data_CSC67.h5 --label sort_pos_simple`

to create an overview plot:

You can also display this plot within `css-overview-gui`. This is useful if you work with *job files* (not part of the tutorial).

The overview plot shows a few problems:

- Units 3 and 8 are artifacts
- Units 5 and 7 look quite similar and should maybe be merged.

We will now use the `css-gui` to fix these problems.

4.2 Using the graphical user interface

Open `css-gui` and load `CSC67` (File, Open). Then find the artifacts in the pull down menu. To move a cluster to the group *Artifacts*, just click on it and press `A`.

To decide whether to merge units 5 and 7 or not, use the Tab *Correlations*:

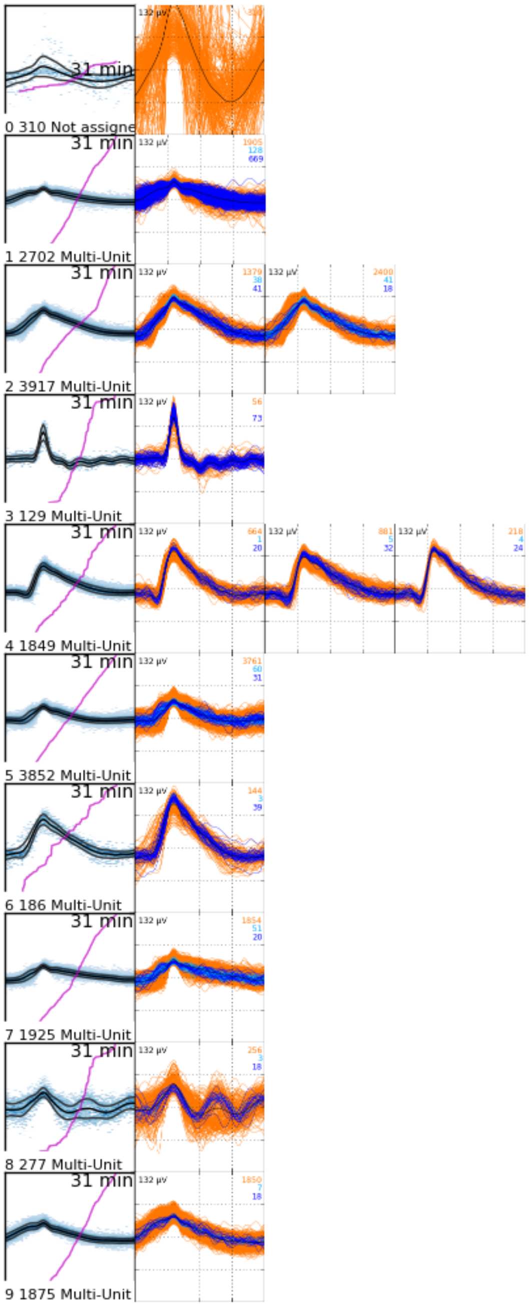

Figure 10: Plot of sorted spikes from CSC67

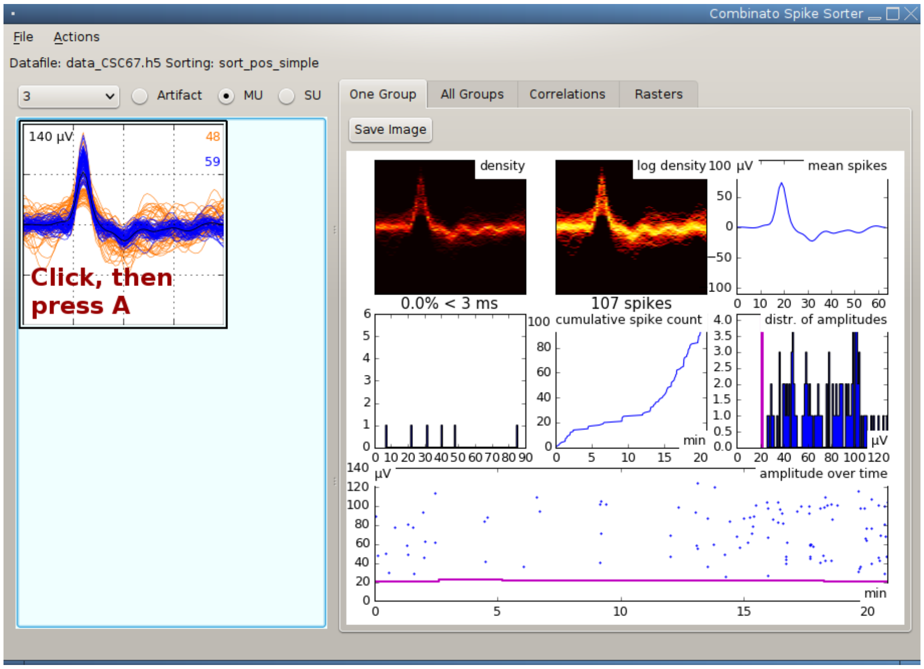

Figure 11: Marking artifacts

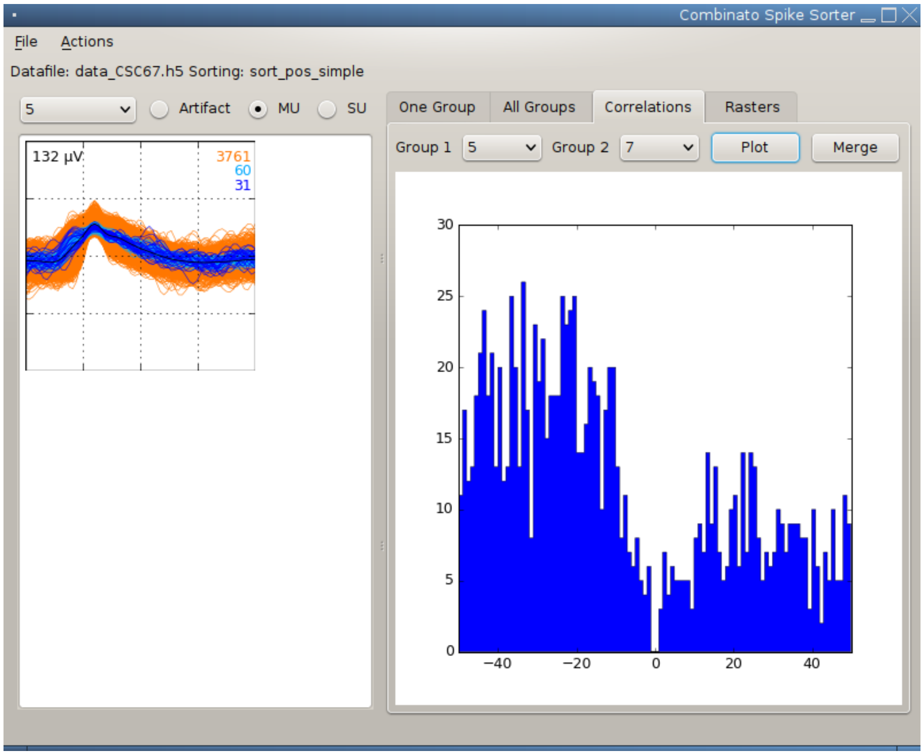

5. Outlook

Combinato offers many possibilities not discussed in the tutorial. Among these are

- Splitting the clustering-combination procedure into individual programs for tuning
- Working with job files created in `css-overview-gui`
- Extracting or sorting only parts of data files, indicated by time ranges
- Excluding time ranges from the sorting procedure (e.g. for known periods of contamination)
- And many more. Also use `--help` as an option to all `css-*` programs!

6. Working with raster plots

One very handy feature of Combinato is that you can create raster plots on-line in `css-gui`. This looks like this:

One unit responds to the tie (both to the image and the written name in German):

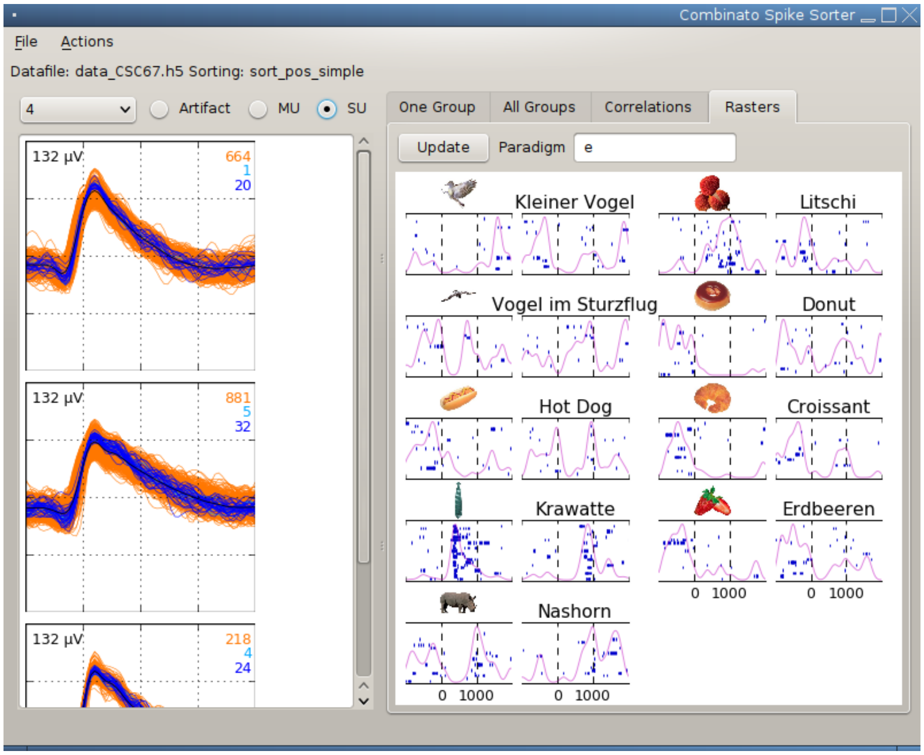

Another unit responds to the strawberry (but not to the written name):

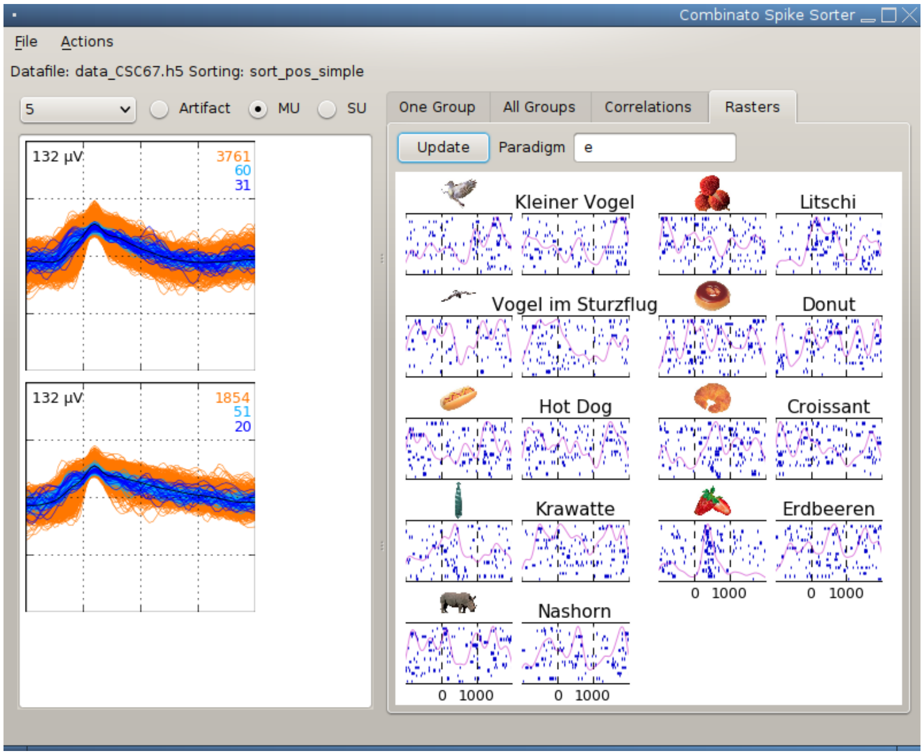

Feel free to contact me (`jonied@posteo.de`) for instructions on how to supply the stimulus material!

Congratulations! You mastered the Combinato tutorial.
